# Low-carbohydrate diets and men's cortisol and testosterone: Systematic review and meta-analysis

**DOI:** 10.1177/02601060221083079

**Published:** 2022-03-07

**Authors:** Joseph Whittaker, Miranda Harris

**Affiliations:** The School of Allied Health and Community, 8709 University of Worcester, Worcester, UK

**Keywords:** Testosterone, cortisol, low carbohydrate diet, ketogenic diet, systematic review, meta-analysis

## Abstract

**Background:** Low-carbohydrate diets may have endocrine effects, although individual studies are conflicting. Therefore, a review was conducted on the effects of low- versus high-carbohydrate diets on men's testosterone and cortisol. **Methods:** The review was registered on PROSPERO (CRD42021255957). The inclusion criteria were: intervention study, healthy adult males, and low-carbohydrate diet: ≤35% carbohydrate. Eight databases were searched from conception to May 2021. Cochrane's risk of bias tool was used for quality assessment. Random-effects, meta-analyses using standardized mean differences and 95% confidence intervals, were performed with Review Manager. Subgroup analyses were conducted for diet duration, protein intake, and exercise duration. **Results:** Twenty-seven studies were included, with a total of 309 participants. Short-term (<3 weeks), low- versus high-carbohydrate diets moderately increased resting cortisol (0.41 [0.16, 0.66], *p* < 0.01). Whereas, long-term (≥3 weeks), low-carbohydrate diets had no consistent effect on resting cortisol. Low- versus high-carbohydrate diets resulted in much higher post-exercise cortisol, after long-duration exercise (≥20 min): 0 h (0.78 [0.47, 1.1], *p* < 0.01), 1 h (0.81 [0.31, 1.31], *p* < 0.01), and 2 h (0.82 [0.33, 1.3], *p* < 0.01). Moderate-protein (<35%), low-carbohydrate diets had no consistent effect on resting total testosterone, however high-protein (≥35%), low-carbohydrate diets greatly decreased resting (−1.08 [−1.67, −0.48], *p* < 0.01) and post-exercise total testosterone (−1.01 [−2, −0.01] *p* = 0.05). **Conclusions:** Resting and post-exercise cortisol increase during the first 3 weeks of a low-carbohydrate diet. Afterwards, resting cortisol appears to return to baseline, whilst post-exercise cortisol remains elevated. High-protein diets cause a large decrease in resting total testosterone (∼5.23 nmol/L).

## Introduction

Testosterone (T) is the primary male sex hormone, and vital for reproductive development and function. Moreover, low endogenous T is associated with an increased risk of chronic disease, including type 2 diabetes (Yao et al., 2018) and cardiovascular disease (Corona et al., 2018). In many respects, cortisol is biochemically opposed to T, as the administration of exogenous cortisol lowers T (Cumming et al., 1983). Also, environmental stressors such as exercise reciprocally increase cortisol and decrease total testosterone (TT) (Brownlee et al., 2005). Similarly, the reverse relationship between cortisol and chronic disease risk is observed, with higher levels being associated with an increased risk of cardiovascular disease mortality (Vogelzangs et al., 2010). The relationship between testosterone and cortisol likely stems from their respective anabolic and catabolic properties.

In recent decades, research has found low-carbohydrate (LC) diets have positive health effects including: weight loss, decreased triglycerides, and increased high-density lipoprotein cholesterol (Dong et al., 2020). Although, increased total cholesterol is a notable exception to this (Dong et al., 2020). Interestingly, a 2021 study found an 8-week LC diet significantly increased TT (+6.43 nmol/L) (Vidić et al., 2021), suggesting LC diets may have endocrine effects. However, the 5.7 kg weight loss during this study, which is well known to increase T (Corona et al., 2013), likely confounded the results. Furthermore, cortisol has a number of glucoregulatory roles (Kuo et al., 2015), and so may also be sensitive to carbohydrate intake. The impact of carbohydrate intake on post-exercise cortisol is of particular interest, as a meta-analysis showed carbohydrate supplements during exercise significantly attenuated the rise in post-exercise cortisol (−124 nmol/L) (Moreira et al., 2007). This suggests carbohydrate intake via diet, may similarly affect post-exercise cortisol. Also, a recent narrative review has suggested macronutrients may affect the anabolic response to exercise (Zamir et al., 2021), which indicates post-exercise T may be subject to dietary influence.

Studies using isocaloric LC diets have shown conflicting effects on T and cortisol (Michalczyk et al., 2019; Zajac et al., 2014), highlighting the need for systematic review. Moreover, previous reviews have only briefly covered the topics of T, cortisol, and LC diets; with none including meta-analyses or statistical investigations of heterogeneity (Kang et al., 2020). Of particular interest is the duration of LC diets, as these diets have an adaptation period of ∼3 weeks (Burke et al., 2017), or longer (Sherrier and Li, 2019). Thus, the effects on steroid hormones may differ pre- and post-adaptation. In addition, LC diets can either be high-protein (HP) or moderate-protein (MP), which may further help to differentiate effects. Therefore, we conducted a systematic review and meta-analysis, with statistical investigations of heterogeneity, on the effects of LC versus high-carbohydrate (HC) diets on men's T and cortisol.

## Methods

The review was registered on PROSPERO (CRD42021255957) (Whittaker and Harris, 2021), and reported according to the PRISMA 2020 checklists (Supplementary Appendix - Tables 1, 2, and 3) (Page et al., 2021; Rethlefsen et al., 2021). The methods of this review are partly based on a previous systematic review by the lead author (Whittaker and Wu, 2021). *The Cochrane Handbook for Systematic Reviews of Interventions* was consulted throughout the review process (Higgins, Thomas, et al., 2021).

### Eligibility criteria

Studies were eligible which met all of the following criteria.

1. Intervention study.
2. Measurement via blood sample of either resting TT; resting cortisol; 0 h, 1 h or 2 h post-exercise cortisol.
3. Healthy adult male participants (18 + years), to minimize variation in steroid hormone metabolism due to age, sex, and disease.
4. A LC diet ≤35% and HC diet >35% carbohydrate intake, measured as a percentage of total energy intake (TEI).
5. A difference in carbohydrate intake between LC and HC diets ≥20% of TEI, to sufficiently distinguish intervention diets on the basis of carbohydrate intake.
6. Duration of intervention diets ≥24 h, to minimize the influence of acute post-prandial variations in steroid hormones.
7. None of the following confounding variables which may affect steroid hormone metabolism: body mass change ≥3 kg (Corona et al., 2013), carbohydrate loading on LC diets (Wilson et al., 2020), exogenous hormones, phytoestrogens (Domínguez-López et al., 2020), or medications besides over-the-counter ones (Schooling et al., 2013).

In the literature, there is no consensus on what carbohydrate intake constitutes a LC diet. The UK government's dietary guidelines recommend ∼55% carbohydrate intake (Public Health England, 2016). Since, we required ≥20% difference in carbohydrate intake between intervention diets, a LC diet was defined as ≤35% carbohydrate intake. TEI rather than grams was used to measure carbohydrate intake, to account for participant differences in energy intake requirements. Randomized and non-randomized studies were included to provide a more comprehensive assessment of the evidence. <3 kg body mass change was deemed acceptable, as very LC diets incur ∼1.4 kg water weight loss (Hall et al., 2016). Studies not reporting body mass change were still eligible, provided that intervention diets were isocaloric, which was pragmatically defined as diets within 15% TEI of each other.

### Search strategy

**Figure 1.**
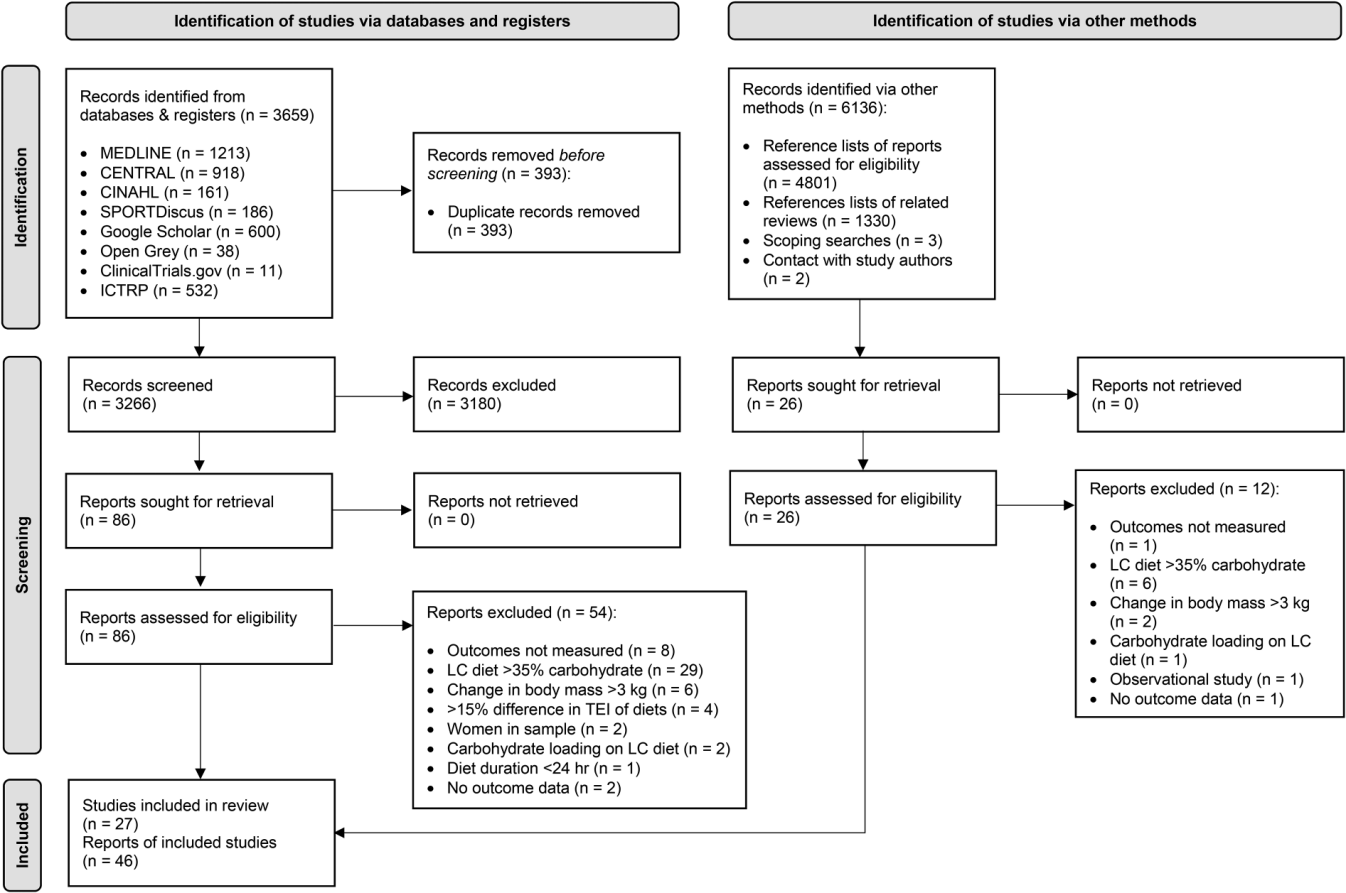
PRISMA study selection flowchart. LC: Low-carbohydrate; TEI: Total energy intake.

Eight databases were searched from conception to 25th May 2021: MEDLINE, CENTRAL, CINAHL, SPORTDiscus, Google Scholar, Open Grey, ClinicalTrials.gov, and ICTRP. The key search terms used were: testosterone, cortisol, low carbohydrate diet, high carbohydrate diet, and their synonyms. Where possible the search filter ‘adult male’ was used. No limits were put on date or language. For full details of the search terms and filters used see: Supplementary Appendix - Table 4. The search, and initial title and abstract screen was done by one author (J.W). Duplicate records were removed by the software: Mendeley Desktop (Mendeley, 2021). Thereafter, both authors independently screened each full text report for eligibility; differences between authors were settled by discussion. References lists of reports selected for full text screening and related reviews were also screened. The search process was documented in a PRISMA 2020 flowchart (Figure 1) (Page et al., 2021).

### Data extraction

Both authors independently extracted study characteristics and outcome data (Supplementary Appendix – Tables 6 and 7, Figure 2). When available, change from baseline data was extracted, to account for participant variation in outcomes. Afterwards, all data was double-checked and any differences resolved by reference to the original study reports. Study authors were contacted to request missing data, and graphical data was extracted using the software: WebPlotDigitizer (Rohatgi, 2021). Additional considerations regarding data extraction unique to individual studies are detailed in: Supplementary Appendix - Section 1.

### Meta-analyses

**Figure 4.**
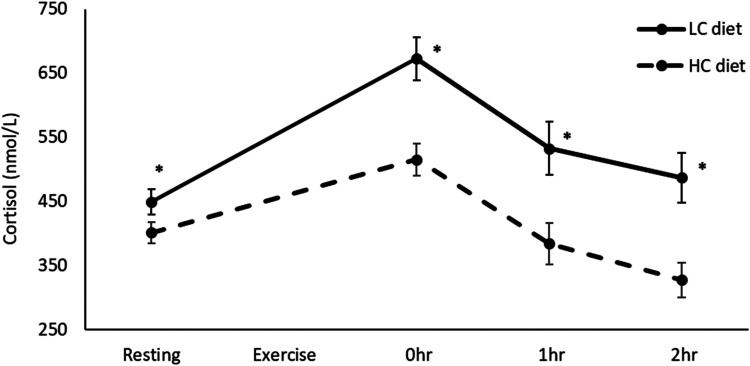
Resting, 0 h, 1 h, and 2 h post-exercise cortisol after long-duration exercise, on short-term LC and HC diets. Resting (15 studies, LC: n = 131, HC: n = 134), 0 h (8 studies, LC: n = 72, HC: n = 75), 1 h (4 studies, LC: n = 41, HC: n = 40), 2 h (3 studies, LC: n = 36, HC: n = 36). Values in mean ± SE. *p < 0.001 compared to HC diet. HC: High-carbohydrate; LC: Low-carbohydrate.

The studies contained a mix of plasma and serum samples with differing assays. Therefore, to account for possible inter-sample variation standardized mean differences (SMD) with 95% confidence intervals (CI) were used. Change score SDs were not used as this would have required variance to be calculated based on assumptions for the majority of studies (Higgins, Eldridge, et al., 2021). The results included parallel and crossover studies, thus to standardize the weighting across study designs and data types, post-intervention standard deviations (SD) were used for all studies (Deeks et al., 2021). The longest follow-up for each study was used. Standard errors (SE) were converted into SDs, subgroups within studies combined, and random-effects, meta-analyses were performed using Review Manager (The Cochrane Collaboration, 2020). Chi^2^ and I^2^ tests were used to measure heterogeneity, with I^2^ ≥30% and Chi^2^ *p* < 0.1 interpreted as evidence of heterogeneity (Deeks et al., 2021). SMDs were interpreted as: 0.2 = small effect, 0.5 = moderate effect, and 0.8 = large effect (Cohen, 1988). A post-hoc meta-analysis was conducted for post-exercise TT, to replicate the analysis for cortisol. Also, cortisol data for short-term LC diets and long-duration exercise was pooled and plotted onto a graph, as these results were homogeneous (Figure 4). Lastly, a post-hoc mean difference was calculated for TT: HP-LC diets using pre- and post-interventions means, to aid with clinical interpretation.

### Subgroup analyses

When heterogeneity was detected, two *a priori* subgroup analyses were conducted. Firstly, on the basis of LC diet duration: long- (≥3 weeks) versus short-term (<3 weeks) LC diets, and if heterogeneity remained unexplained, secondly on the basis of LC diet protein intake: MP (<35% protein) versus HP (≥35% protein) LC diets. In one outcome (0 h post-exercise cortisol) heterogeneity remained unexplained after both *a priori* subgroup analyses, therefore one post-hoc subgroup analysis was conducted on the basis of exercise duration: long- (≥20 min) versus short-duration (<20 min) exercise. Statistical differences between subgroups were investigated using Chi^2^ and I^2^ tests, with I^2^ ≥40% and Chi^2^ *p* < 0.05 interpreted as evidence of subgroup effects (Deeks et al., 2021).

### Sensitivity analyses

**Figure 2.**
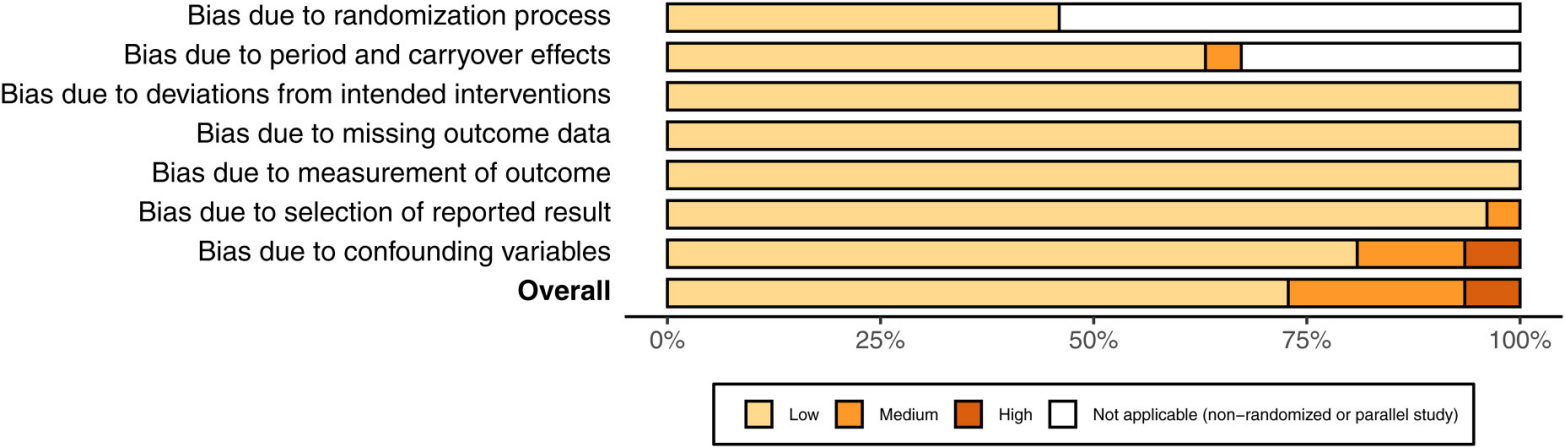
Risk of bias of included studies.

Sensitivity analyses were run on all meta- and subgroup analyses, besides the three subgroup analyses that did not explain heterogeneity (Supplementary Appendix – Figures 2b, 2c, and 2g). An *a priori* sensitivity analysis was conducted by excluding each study in turn, and running the analyses again. Four post-hoc sensitivity analyses were conducted: exclusion of high and medium bias studies, exclusion of non-randomized studies, exclusion of studies using carbohydrate supplements during exercise on HC diets, and using alternative HC diets from studies with two eligible HC diets (Supplementary Appendix – Table 9).

### Risk of bias assessment

Cochrane's risk of bias tool for randomized studies was used for quality assessment (Higgins, Li, et al., 2021; Sterne et al., 2019). For non-randomized studies, the randomization questions were omitted. An additional bias domain was added for bias due to confounding variables. Both authors independently conducted the quality assessment, and differences were settled by discussion. Review and study level bias figures were produced using Cochrane's software: Robvis (Figure 2, Supplementary Appendix – Figure 1) (McGuinness and Higgins, 2021).

### Certainty of evidence assessment

**Figure 3.**
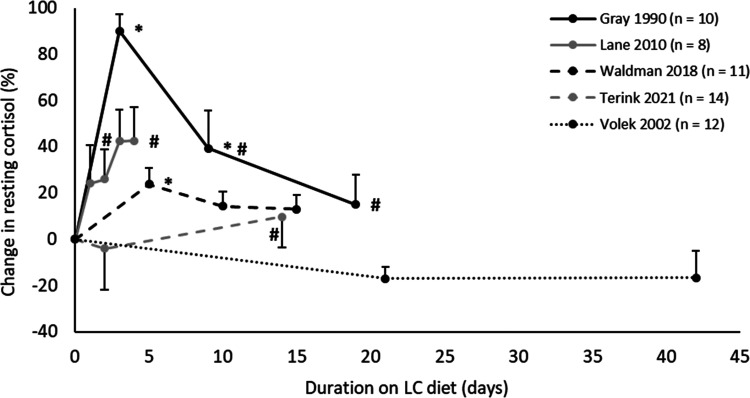
Percentage change in resting cortisol over time on LC diets, in studies with repeated measurements (HC diet = 100%). Values in mean ± SE. *p < 0.05 compared to HC diet. ^#^*p* < 0.05 compared to first measurement on LC diet. HC: High-carbohydrate; LC: Low-carbohydrate.

For each of the key findings, a GRADE assessment was conducted (Table 1) (Schünemann et al., 2013). Both authors independently conducted the assessments, and differences were settled by discussion. Publication bias was assessed as part of the GRADE assessments, and was based on visual inspection of funnel plots, conflicts of interests, and number of studies excluded for missing outcome data. Statistical tests for funnel plot asymmetry were not undertaken, as the included studies had similar SEs (Sterne et al., 2011). Funnel plots were generated using Review Manager (Supplementary Appendix – Figure 3) (The Cochrane Collaboration, 2020).

**Table 1.** Summary of findings.

Outcome & subgroup^a^	Participants(studies)	SMD [95% CI]	P-value (Z test)	I^2^ (%)	P-value (Chi^2^ test)	Interpretation	Certainty of evidence (GRADE rating)
Cortisol, resting							
All studies	245 (22)	0.18 [−0.06, 0.42]	0.15	32	0.08		
Long-term LC diets (MP-LC diets only)	90 (7)	−0.28 [−0.7, 0.15]	0.2	36	0.15	Long-term, MP-LC vs HC diets neither increase nor decrease resting cortisol.	**Moderate** ⊕⊕⊕⊝ Downgraded due to unexplained heterogeneity
Short-term LC diets	155 (15)	0.41 [0.16, 0.66]	<0.01	0	0.63	Short-term LC vs HC diets moderately increase resting cortisol.	**High** ⊕⊕⊕⊕
Test for subgroup differences				86.7	<0.01		
Cortisol, 0 h post-exercise							
All studies	129 (12)	0.58 [0.17, 0.99]	<0.01	50	0.02		
Long-duration exercise	112 (10)	0.78 [0.47, 1.1]	<0.01	0	0.51	LC vs HC diets result in much higher 0 h post-exercise cortisol, after long-duration exercise.	**Moderate** ⊕⊕⊕⊝ Downgraded due to indirectness (only includes two long-term LC diet studies)^b^
Short-duration exercise	17 (2)	−0.67 [−1.37, 0.03]	0.06	0	0.78	LC vs HC diets result in much lower 0 h post-exercise cortisol, after short-duration exercise.	**Low** ⊕⊕⊝⊝ Downgraded due to imprecision (small sample size and *p* > 0.05)
Test for subgroup differences				92.8	<0.01		
Cortisol, 1 h post-exercise							
All studies (long-duration exercise only)	55 (5)	0.81 [0.31, 1.31]	<0.01	24	0.26	LC vs HC diets result in much higher 1 h post-exercise cortisol, after long-duration exercise.	**Moderate** ⊕⊕⊕⊝ Downgraded due to indirectness (only includes one long-term LC diet study)^b^
Cortisol, 2 h post-exercise							
All studies (short-term, MP-LC diets and long-duration exercise only)	36 (3)	0.82 [0.33, 1.3]	<0.01	0	0.79	Short-term, MP-LC vs HC diets result in much higher 2 h post-exercise cortisol, after long-duration exercise.	**Moderate** ⊕⊕⊕⊝ Downgraded due imprecision (small sample size)
Total testosterone, resting							
All studies	155 (13)	−0.48 [−0.87, −0.09]	0.01	49	0.02		
MP-LC diets	129 (10)	−0.31 [−0.74, 0.13]	0.17	48	0.04	MP-LC vs HC diets neither increase nor decrease resting total testosterone.	**Low** ⊕⊕⊝⊝ Downgraded due to indirectness (only includes two short-term LC diet studies)^b^ and risk of bias (randomized studies *only*: SMD = −0.79, *p* < 0.01, I^2^ = 0%)
HP-LC diets (short-term LC diets only)	26 (3)	−1.08 [−1.67, −0.48]	<0.01	0	0.85	Short-term HP-LC vs HC diets greatly decrease resting total testosterone.	**Moderate** ⊕⊕⊕⊝ Downgraded due to imprecision (small sample size)
Test for subgroup differences				76.5	0.04		
Total testosterone, 0 h post-exercise							
All studies	28 (3)	−0.03 [−0.95, 0.89]	0.95	65	0.06		
Long-term LC diets (MP-LC diets only)	19 (2)	0.44 [−0.21, 1.09]	0.18	0	0.9	Long-term, MP-LC vs HC diets result in higher 0 h post-exercise total testosterone.	**Very low** ⊕⊝⊝⊝ Downgraded due to indirectness (only includes one long- and one short-duration exercise study)^c^ and imprecision (small sample size and *p* > 0.05)
Short-term LC diets (HP-LC diets and long-duration exercise only)	9 (1)	−1.01 [−2, −0.01]	0.05	NA	NA	Short-term, HP-LC vs HC diets result in much lower 0 h post-exercise total testosterone, after long-duration exercise.	**Low** ⊕⊕⊝⊝ Downgraded due to imprecision (small sample size and *p* = 0.05)
Test for subgroup differences				82.5	0.02		

^a^ Long-term (≥3 weeks), short-term (<3 weeks), long-duration exercise (≥20 min), short-duration exercise (<20 min), MP (<35% protein), HP (≥35% protein).

^b^ Indirect evidence drawn from long or short LC diets, to support a conclusion about LC diets in general.

^c^ Indirect evidence drawn from long- or short-duration exercise, to support a conclusion about exercise in general.

CI: confidence interval; HC: high-carbohydrate; HP: high-protein; LC: low-carbohydrate; MP: moderate-protein; SMD: standardized mean difference.

## Results

### Study selection

The initial title and abstract screen covered 9402 total records, 3,266 from databases and 6,136 from other sources (Figure 1). Next, 112 reports encompassing 78 studies were selected for full text screening, of which 27 studies encompassed within 46 reports were selected. For a list of key excluded studies see: Supplementary Appendix – Table 5.

### Characteristics of included studies

There was a total of 309 participants (mean ± SD), age: 27.3 ± 4.7 (n = 309), body mass: 78.6 ± 7.1 kg (n = 303), and BMI: 24.8 ± 1.6 (n = 246) (Supplementary Appendix – Table 6). 95% of the participants were physically active during dietary interventions, and post-exercise cortisol measurements were all taken after aerobic exercise tests. Exercise tests ranged from 15 – 232 min, with intensities from ∼60 – 100% of maximal oxygen uptake. Dietary interventions ranged from 2 days – 8 weeks; and mean differences in LC versus HC diets were, body mass change: −1.4 kg (LC: −1.5; HC: −0.1; n = 222), energy intake: 8 kcal (LC: 2,873; HC: 2,866; n = 275), and carbohydrate intake: −46% of TEI (LC: 12%; HC: 58%; n = 309). Also, LC versus HC diets were higher in fat, protein, and cholesterol; whilst lower in fibre and sugar (Supplementary Appendix – Table 7).

### Risk of bias

Twenty-one studies were low, five medium, and one high risk of bias; of which 12 were randomized and 15 non-randomized (Figure 2, Supplementary Appendix – Figure 1). For further details on the reasons for bias ratings see: Supplementary Appendix – Table 8. No conflicts of interests for study authors were identified, funnel plots showed no clear asymmetry (Supplementary Appendix – Figure 3), and the number of studies excluded for missing outcome data was two (Supplementary Appendix – Table 5). Thus, publication bias was unlikely to have affected the results.

### Resting cortisol

There was no overall effect of LC versus HC diets on resting cortisol (SMD = 0.18, *p* = 0.15) (Table 1). However, when studies were split into long- (≥3 weeks) versus short-term (<3 weeks) LC diets, there was a moderate increase in resting cortisol on short-term LC diets (SMD = 0.41, *p* < 0.01). Whereas, long-term LC diets showed no consistent effect on resting cortisol (SMD = −0.28, *p* = 0.2). The trend of LC diets increasing resting cortisol in the short-term, but returning to baseline levels in the long-term, can be seen within individual studies measuring cortisol over time, with four out five studies showing this trend (Figure 3).

### Post-exercise cortisol

0 h post-exercise cortisol was much higher on LC versus HC diets (SMD = 0.58, *p* < 0.01) (Table 1). Subgroup analysis revealed the effect was stronger after long-duration exercise (≥20 min) (SMD = 0.78, *p* < 0.01), and reversed after short-duration exercise (<20 min) (SMD = −0.67, *p* = 0.06). 1 h and 2 h post-exercise cortisol were much higher on LC versus HC diets, after long-duration exercise (1 h: SMD = 0.81, *p* < 0.01; 2 h: SMD = 0.82, *p* < 0.01). The effects of short-term LC diets on resting and post-exercise cortisol after long-duration exercise were homogeneous, and show cortisol increasing more sharply during exercise and remaining elevated post-exercise (Figure 4).

### Resting total testosterone

The overall results for resting TT showed a significant decrease on LC versus HC diets (SMD = −0.48, *p* = 0.01) (Table 1). However, subgroup analyses revealed this effect to be limited to HP-LC diets, which yielded a very large decrease in TT (SMD = −1.08, *p* < 0.01; ∼5.23 nmol/L), albeit in a small sample (n = 26). MP-LC diets had no consistent effect on TT (SMD = −0.31, *p* = 0.17).

### Post-exercise total testosterone

There was no overall effect of LC versus HC diets on 0 h post-exercise TT (SMD = −0.03, *p* = 0.95) (Table 1). However, subgroup analysis showed 0 h post-exercise was non-significantly higher on long-term LC versus HC diets (SMD = 0.44, *p* = 0.18), and much lower on short-term LC versus HC diets (SMD = −1.01, *p* = 0.05); although both subgroups had small samples (n = 19, n = 9). The long- and short-term diet subgroups contained *solely* MP- and HP-LC diets, respectively. Meaning the observed subgroup effects could either be attributable to diet duration or protein intake.

### Sensitivity analyses

The results were robust to sensitivity analyses, besides two exceptions (Supplementary Appendix – Table 9). Firstly, excluding non-randomized studies for TT: MP-LC diets, produced a stronger, more significant effect, with reduced heterogeneity (SMD = −0.79 [−1.37, −0.22], *p* < 0.01; I^2^ = 0%, *p* = 0.69). Secondly, excluding studies using carbohydrate supplements during exercise on HC diets, produced a weaker effect for 0 h and 1 h post-exercise cortisol (0 h, SMD = 0.41 [−0.01, 0.83]; 1 h, SMD = 0.6 [0.11, 1.09]).

## Discussion

### Resting cortisol

The increase in resting cortisol on short but not long-term LC diets, is likely tied to glucocorticoids’ roles in glucose homeostasis. Cortisol, glucagon, and gluconeogenesis all increase on short, but not long-term LC diets (Bisschop et al., 2000; Muller et al., 1971; Webster et al., 2016). As glucocorticoids increase gluconeogenesis (Kuo et al., 2015), the initial rise in cortisol may be partly responsible for a transient increase in gluconeogenesis, on short LC diets. Additionally, cortisol may rise to spare glucose for brain function, as the brain cannot significantly use fatty acids for fuel. Glucocorticoids inhibit glucose uptake and oxidation in adipose tissue and skeletal muscle, thereby conserving glucose for brain function (Kuo et al., 2015). In contrast, endogenous ketone production increases sharply over the first 3 weeks of a very LC diet (Vidić et al., 2021), and ketones can be used for fuel by the brain (Zhang et al., 2013). Thus, when ketones replace glucose for the majority of brain fuel, cortisol's glucose sparing effects are not needed, and thus levels may return to baseline.

### Post-exercise cortisol

The results showed the increase in cortisol during exercise was greater on LC diets. Moreover, this effect appears to persist post-adaptation to a LC diet, although somewhat lessened (Terink et al., 2021). Interestingly, the rise in post-exercise cortisol was reduced in studies using carbohydrate supplements during exercise on HC diets (Supplementary Appendix – Table 9). This is supported by another meta-analysis which found post-exercise cortisol was significantly attenuated by carbohydrate supplementation during exercise (Moreira et al., 2007). Thus, it appears the rise in cortisol during exercise is increased during times of low carbohydrate availability. There are three possible, complementary explanations for this. Firstly, as glycogen stores are partially depleted on a LC diet (Webster et al., 2016), cortisol may increase more sharply on LC diets to facilitate increased gluconeogenesis during exercise. Secondly, fat oxidation is higher on LC versus HC diets during exercise (Webster et al., 2016), and thus cortisol may increase to facilitate increased fat oxidation via inducing lipolysis in adipose tissue (Djurhuus et al., 2002; Kuo et al., 2015). Thirdly, exercise upregulates skeletal muscle glucose uptake (Evans et al., 2019), thus cortisol may increase to preserve glucose for brain function.

Additionally, the results showed post-exercise cortisol was lower after short, intense exercise on LC versus HC diets. This subgroup analysis was post-hoc, and thus more likely a false-positive finding. However, the qualitative interaction observed suggests differential effects. Short, intense exercise predominantly uses glucose for fuel, whereas long-duration exercise increasingly relies on fatty acids (Purdom et al., 2018). Also, carbohydrate supplementation only attenuates cortisol during long-duration exercise (Moreira et al., 2007). Therefore, on LC diets muscle glycogen may be sufficient for short exercise (Cipryan et al., 2018), whereas for long exercise lipolysis stimulated via cortisol may be increasingly required.

### Resting testosterone

MP-LC diets had no consistent effect on resting TT, however HP-LC diets caused a large decrease in resting TT. For context, mean TT for a comparably aged population (27 years) is 14 nmol/L (Kelsey et al., 2014), thus −5.23 nmol/L represents a 37% decrease. Protein intakes ≥35% may outstrip the urea cycle's capacity to convert nitrogen derived from amino acid catabolism into urea, leading to hyperammonaemia and its toxic effects (Bilsborough and Mann, 2006). T has been shown to suppress the urea cycle (Lam et al., 2017), whilst glucocorticoids upregulate the urea cycle (Okun et al., 2015). Notably, the largest decrease in resting cortisol was on the longest and best-controlled HP-LC diet study (Supplementary Appendix – Figure 2a). Thus, the decrease in T and increase in cortisol on HP diets, may serve to upregulate the urea cycle and increase nitrogen excretion, thereby limiting the adverse effects of excess protein consumption.

### Post-exercise testosterone

The results showed post-exercise TT was higher on long-term MP-LC diets, and lower on short-term HP-LC diets. The finding that HP-LC diets caused a large decrease in resting TT, whilst long-term LC diets had no effect on resting TT, suggests the observed subgroup effects in post-exercise TT are explained by protein intake rather than diet duration. HP intakes may depress post-exercise TT to maintain upregulation of the urea cycle and increased nitrogen excretion, as previously discussed (Discussion: Resting testosterone). The finding that long-term LC diets increased post-exercise TT, may be explained by the increase in blood cholesterol on LC diets (Dong et al., 2020), providing greater substrate for T production, which is utilized in times of increased anabolic signalling, such as during exercise (Pasiakos, 2012).

### Practical implications

The increase in cortisol during the first 3 weeks of a LC diet is likely part of the adaption process to such diets, and thus may not represent a pathological state. The results indicate cortisol returns to baseline levels after ∼3 weeks, suggesting cardiovascular disease risk is not elevated by higher cortisol on long LC diets. However, the effects of long-term LC diets on cardiovascular disease and all-cause mortality, as measured by other methods, are uncertain. Observational studies have found an increase in all-cause mortality on long-term LC diets (Noto et al., 2013), whilst interventional studies have shown improvements in cardiovascular disease biomarkers (Dong et al., 2020). Additional research on the effects of long-term LC diets is desirable, particularly as these diets have risen in popularity over recent years.

The higher increase in cortisol during exercise on LC versus HC diets appears to persist post-adaptation. Classically, cortisol is thought to have immunosuppressive effects, however in spite of elevated post-exercise cortisol, LC diets do not appear overtly immunosuppressive, according to other immune-markers (Shaw et al., 2021). The potential immunosuppressive effects of higher post-exercise cortisol may be exacerbated in athletes undergoing high volume training, and some caution may be advisable, until further research is undertaken.

The large decrease in resting and post-exercise TT on LC-HP diets may only occur on diets that outstrip the urea cycle's capacity to synthesize urea, as there were no clear adverse endocrine effects for LC diets using 30–31% protein intake (Supplementary Appendix – Table 7 and Figure 2). In practise, most free-living LC diets will fall below the urea cycle capacity threshold (≤35% protein), as population protein intakes are stable at 15–17% (Cohen et al., 2015), likely due to a protein-specific appetite mechanism (Leidy et al., 2015). However, one can find articles online advocating protein intakes ≤35%, which if followed precisely, may lead to adverse endocrine effects, particularly in individuals with lower rates of maximal urea synthesis (Bilsborough and Mann, 2006).

The higher post-exercise TT on MP-LC diets may signal an increased anabolic response to exercise, which would be advantageous, particularly in individuals with strength, power, or hypertrophy goals. Relatedly, another systematic review found that whilst absolute strength and power were unchanged by LC diets, the decrease in body fat on LC diets resulted in an improved strength/power to bodyweight ratio (Kang et al., 2020). However, the finding that LC diets increase post-exercise T should be taken with caution, as although the direction of effects was consistent, due to the small sample size, the p-value remained high. Ideally, this finding should be viewed as hypothesis generating, to be confirmed by future research.

### Limitations

Firstly, there was unexplained heterogeneity in both resting cortisol and TT on long-term LC diets. The three studies which reported micronutrients showed diverse intakes on LC diets (Supplementary Appendix – Table 7), suggesting a possible source of the heterogeneity. However, due to lack of study data, this was unable to be explored as a source of heterogeneity. Secondly, the heterogeneity present in TT, long-term LC diets may be due to methodological diversity. The results for randomized studies *only*, showed a decrease in TT on MP-LC diets (SMD = −0.79, *p* < 0.01), suggesting study design may have impacted the results. Thirdly, there were differences in exercise regimes and carbohydrate intakes on LC diets (Supplementary Appendix - Tables 6 and 7). However, due to the low number of studies and risk of data dredging, these factors were not explored as possible sources of heterogeneity. This issue brings up the wider debate about ‘lumping versus splitting’ in meta-analyses (Thomas et al., 2021). Dietary studies are typically dissimilar to each other in at least one important respect, and meta-analyses attempt to estimate effects across similar, but not identical studies. As readers tend to make judgements about sets of studies regardless, it is arguably preferable to investigate such differences using statistical methods, as this is more objective. The approach of this review was to pool studies similar in major aspects (diet), but dissimilar in other aspects (duration, protein intake), and address the dissimilarities using subgroup analyses.

An issue of particular note is that the finding that HP-LC diets decreased T, may be subject to residual confounding. Per calorie, protein is more satiating than fat or carbohydrate, thus HP diets commonly cause a decrease in energy intake via increased satiety (Paddon-Jones et al., 2008); and low energy intakes are known to decrease T (Henning et al., 2014). Of the three HP diet studies, two studies provided the participants with isocaloric diets and either reported no significant changes in bodyweight (Anderson et al., 1987), or did not mention it (Langfort et al., 2001). In the third study, participants constructed their diets themselves, based on guidance from the researchers (Jaffe, 2013). Reported daily energy intakes were 23 kcal, and bodyweight 0.1 kg, lower on LC versus HC diets. Thus, it appears that energy intake was not a confounding variable in the HP diet studies, although the possibility of dietary misreporting remains in the third study.

The review had a number of strengths. Firstly, the overall sample was homogenous, being: male, young to middle-aged adults, healthy, non-obese, and physically active. However, this also means the findings are harder to generalize to other populations. Sex differences have been found in carbohydrate and lipid metabolism during exercise (Purdom et al., 2018), which may extend to post-exercise cortisol. Secondly, the majority of data was from low bias studies (73%) (Figure 1), and the results were robust to differences between randomized and non-randomized studies, besides one subgroup (TT: MP-LC) (Supplementary Appendix – Table 9). Finally, the extensive search process covering 9402 records likely identified all eligible studies (Figure 1), including studies from grey literature (Supplementary Appendix – Table 6).

### Conclusions

This review found an increase in resting and post-exercise cortisol on short-term LC diets (<3 weeks). In addition, the results indicate resting cortisol levels return to baseline after ∼3 weeks on a LC diet, whilst post-exercise cortisol remains elevated. However, due to the low number of studies and unexplained heterogeneity in long-term LC diets, further research is needed to confirm the latter effects. MP-LC diets appear to have no effect on resting TT, although the decrease in resting TT on randomized MP-LC studies, highlights the need for further randomized controlled trials. Finally, HP-LC diets caused a large decrease in resting TT, suggesting individuals consuming such diets may need to be cautious about adverse endocrine effects.

## Supplemental Material

sj-pdf-1-nah-10.1177_02601060221083079 - Supplemental material for Low-carbohydrate diets and men's cortisol and testosterone: Systematic review and meta-analysis Click here for additional data file. Supplemental material, sj-pdf-1-nah-10.1177_02601060221083079 for Low-carbohydrate diets and men's cortisol and testosterone: Systematic review and meta-analysis by Joseph Whittaker and Miranda Harris in Nutrition and Health

## List of Abbreviations

CI: confidence interval
LC: low-carbohydrate
HC: high-carbohydrate
HP: high- protein
MP: moderate-protein
SD: standard deviation
SE: standard error
SMD: standardized mean difference
TEI: total energy intake
T: testosterone
TT: total testosterone
