# Supplementary material for: Low-carbohydrate diets and men's cortisol and testosterone: Systematic review and meta-analysis
Source: Nutr Health. 2022 Mar 7;28(4):543–54. doi: 10.1177/02601060221083079 (PMC9716400; doi:10.1177/02601060221083079)
Supplement: sj-pdf-1-nah-10.1177_02601060221083079 - Supplemental material for Low-carbohydrate diets and men's cortisol and testosterone: Systematic review and meta-analysis [file sj-pdf-1-nah-10.1177_02601060221083079.pdf]

# Appendix

## Table of Contents

**Tables 1 - 3: PRISMA checklists** ... **2**
Table 1: PRISMA 2020 main checklist ... 2
Table 2: PRISMA 2020 abstract checklist ... 6
Table 3: PRISMA 2021 search extension checklist ... 7
**Table 4: Database searches** ... **9**
**Section 1: Additional considerations regarding data extraction** ... **11**
**Table 5: Excluded studies** ... **12**
**Table 6: Characteristics of included studies** ... **14**
**Table 7: Characteristics of study diets** ... **20**
**Figure 1: Risk of bias ratings for studies** ... **23**
**Table 8: Reasons for medium and high bias ratings** ... **24**
**Figure 2: Forest plots of meta-analyses** ... **25**
Figure 2a: Cortisol, resting, long- vs short-term LC diets ... 25
Figure 2b: Cortisol, 0 hr post-exercise, long- vs short-term LC diets ... 26
Figure 2c: Cortisol, 0 hr post-exercise, MP- vs HP-LC diets ... 27
Figure 2d: Cortisol, 0 hr post-exercise, long- vs short-duration exercise ... 28
Figure 2e: Cortisol, 1 hr post-exercise ... 29
Figure 2f: Cortisol, 2 hr post-exercise ... 29
Figure 2g: Total testosterone, resting, long- vs short-term LC diets ... 30
Figure 2h: Total testosterone, resting, MP- vs HP-LC diets ... 31
Figure 2i: Total testosterone, 0 hr post-exercise, long- vs short-term diets ... 32
**Figure 3: Funnel plots** ... **33**
Figure 3a: Cortisol, resting, long- vs short-term LC diets ... 33
Figure 3b: Cortisol, 0 hr post-exercise, long- vs short-duration exercise ... 34
Figure 3c: Cortisol, 1 hr post-exercise ... 35
Figure 3d: Cortisol, 2 hr post-exercise ... 35
Figure 3e: Total testosterone, resting, MP- vs HP-LC diets ... 36
Figure 3f: Total testosterone, 0 hr post-exercise, long- vs short-term LC diets ... 37
**Table 9: Sensitivity analyses** ... **38**
**References** ... **45**

---

Abbreviations: BMI: body mass index; CI: confidence interval; HC: high-carbohydrate; HP: high-protein; LC: low-carbohydrate; MP: moderate-protein; NA: not applicable; ND: no data; SD: standard deviation; SE: standard error; SMD: standardized mean difference; $VO_{2max}$: maximal oxygen uptake.

**Table 1.** PRISMA 2020 main checklist.

| Topic | No. | Item | Location reported (or details) |
|---|---|---|---|
| **TITLE** | | | |
| **Title** | 1 | Identify the report as a systematic review. | Title |
| **INTRODUCTION** | | | |
| **Rationale** | 3[a] | Describe the rationale for the review in the context of existing knowledge. | Main text – Section 1, 2nd and 3rd paragraphs |
| **Objectives** | 4 | Provide an explicit statement of the objective(s) or question(s) the review addresses. | Main text – Section 1, 3rd paragraph |
| **METHODS** | | | |
| **Eligibility criteria** | 5 | Specify the inclusion and exclusion criteria for the review and how studies were grouped for the syntheses. | Main text – Section 2.1 |
| **Information sources** | 6 | Specify all databases, registers, websites, organizations, reference lists and other sources searched or consulted to identify studies. Specify the date when each source was last searched or consulted. | Main text – Section 2.2, Appendix – Table 4 |
| **Search strategy** | 7 | Present the full search strategies for all databases, registers and websites, including any filters and limits used. | Appendix - Table 4 |
| **Selection process** | 8 | Specify the methods used to decide whether a study met the inclusion criteria of the review, including how many reviewers screened each record and each report retrieved, whether they worked independently, and if applicable, details of automation tools used in the process. | Main text – Section 2.2, |
| **Data collection process** | 9 | Specify the methods used to collect data from reports, including how many reviewers collected data from each report, whether they worked independently, any processes for obtaining or confirming data from study investigators, and if applicable, details of automation tools used in the process. | Main text – Section 2.3 |
| **Data items** | 10a | List and define all outcomes for which data were sought. Specify whether all results that were compatible with each outcome domain in each study were sought (e.g. for all measures, time points, analyses), and if not, the methods used to decide which results to collect. | Main text – Sections 2.1 and 2.3, Appendix – Section 1, all time points extracted |
| | 10b | List and define all other variables for which data were sought (e.g. participant and intervention characteristics, funding sources). Describe any assumptions made about any missing or unclear information. | Appendix – Section 1, Tables 6 and 7, Figure 2 |
| **Study risk of bias assessment** | 11 | Specify the methods used to assess risk of bias in the included studies, including details of the tool(s) used, how many reviewers assessed each study and whether they worked independently, and if applicable, details of automation tools used in the process. | Main text – Section 2.7 |

| Topic | No. | Item | Location reported (or details) |
|---|---|---|---|
| **Effect measures** | 12 | Specify for each outcome the effect measure(s) (e.g. risk ratio, mean difference) used in the synthesis or presentation of results. | Main text – Section 2.4 |
| **Synthesis methods** | 13a | Describe the processes used to decide which studies were eligible for each synthesis (e.g. tabulating the study intervention characteristics and comparing against the planned groups for each synthesis (item 5)). | Main text – Section 2.4 |
| | 13b | Describe any methods required to prepare the data for presentation or synthesis, such as handling of missing summary statistics, or data conversions. | Main text – Section 2.4 |
| | 13c | Describe any methods used to tabulate or visually display results of individual studies and syntheses. | Main text – Section 2.4 |
| | 13d | Describe any methods used to synthesize results and provide a rationale for the choice(s). If meta-analysis was performed, describe the model(s), method(s) to identify the presence and extent of statistical heterogeneity, and software package(s) used. | Main text – Section 2.4 |
| | 13e | Describe any methods used to explore possible causes of heterogeneity among study results (e.g. subgroup analysis, meta-regression). | Main text – Section 2.5 |
| | 13f | Describe any sensitivity analyses conducted to assess robustness of the synthesized results. | Main text – Section 2.6 |
| **Reporting bias assessment** | 14 | Describe any methods used to assess risk of bias due to missing results in a synthesis (arising from reporting biases). | Main text – Section 2.7 |
| **Certainty assessment** | 15 | Describe any methods used to assess certainty (or confidence) in the body of evidence for an outcome. | Main text – Section 2.8 |
| **RESULTS** | | | |
| **Study selection** | 16a | Describe the results of the search and selection process, from the number of records identified in the search to the number of studies included in the review, ideally using a flow diagram. | Main text – Figure 1 |
| | 16b | Cite studies that might appear to meet the inclusion criteria, but which were excluded, and explain why they were excluded. | Appendix - Table 5 |
| **Study characteristics** | 17 | Cite each included study and present its characteristics. | Appendix - Tables 6 and 7 |
| **Risk of bias in studies** | 18 | Present assessments of risk of bias for each included study. | Appendix - Figure 1 |

| Topic | No. | Item | Location reported (or details) |
|---|---|---|---|
| **Results of individual studies** | 19 | For all outcomes, present, for each study: (a) summary statistics for each group (where appropriate) and (b) an effect estimate and its precision (e.g. confidence/credible interval), ideally using structured tables or plots. | Appendix - Figure 2 |
| **Results of syntheses** | 20a | For each synthesis, briefly summarize the characteristics and risk of bias among contributing studies. | Main text –Table 1, Appendix - Figure 2 |
| | 20b | Present results of all statistical syntheses conducted. If meta-analysis was done, present for each the summary estimate and its precision (e.g. confidence/credible interval) and measures of statistical heterogeneity. If comparing groups, describe the direction of the effect. | Main text – Figure 4, Appendix - Figure 2 |
| | 20c | Present results of all investigations of possible causes of heterogeneity among study results. | Appendix – Figure 2 |
| | 20d | Present results of all sensitivity analyses conducted to assess the robustness of the synthesized results. | Appendix – Table 9 |
| **Reporting biases** | 21 | Present assessments of risk of bias due to missing results (arising from reporting biases) for each synthesis assessed. | Main text – Section 3.3, Appendix – Figure 3 |
| **Certainty of evidence** | 22 | Present assessments of certainty (or confidence) in the body of evidence for each outcome assessed. | Main text – Table 1 |
| **DISCUSSION** | | | |
| **Discussion** | 23a | Provide a general interpretation of the results in the context of other evidence. | Main text – Sections 4.1, 4.2, 4.3, 4.4, and 4.5. |
| | 23b | Discuss any limitations of the evidence included in the review. | Main text – Section 4.6 |
| | 23c | Discuss any limitations of the review processes used. | Main text – Section 4.6 |
| | 23d | Discuss implications of the results for practice, policy, and future research. | Main text – Sections 4.5 and 4.7 |
| **OTHER INFORMATION** | | | |
| **Registration and protocol** | 24a | Provide registration information for the review, including register name and registration number, or state that the review was not registered. | Main text – Section 2 |
| | 24b | Indicate where the review protocol can be accessed, or state that a protocol was not prepared. | Main text – Section 2 |
| | 24c | Describe and explain any amendments to information provided at registration or in the protocol. | PROSPERO record (Whittaker and Harris, 2021) |
| **Support** | 25 | Describe sources of financial or non-financial support for the review, and the role of the funders or sponsors in the review. | Main text – Funding |

| Topic | No. | Item | Location reported (or details) |
|---|---|---|---|
| **Competing interests** | 26 | Declare any competing interests of review authors. | Main text – Declaration of competing interest |
| **Availability of data, code and other materials** | 27 | Report which of the following are publicly available and where they can be found: | |
| | | Template data collection forms | Available on request |
| | | Data extracted from included studies | Appendix – Tables 6 and 7, and Figure 2 |
| | | Data used for all analyses | Appendix - Figure 2, other data available on request |
| | | Analytic code | Chapter 10: S1, *Cochrane handbook for systematic reviews of interventions* (Deeks and Higgins, 2021) |
| | | Any other materials used in the review | Available on request |

[a] Duplicate entries across PRISMA checklists were removed, but the original numbering was retained.

**Table 2.** PRISMA 2020 abstract checklist.

| Topic | No. | Item | Reported? |
|---|---|---|---|
| **BACKGROUND** | | | |
| **Objectives** | 2[a] | Provide an explicit statement of the main objective(s) or question(s) the review addresses. | Yes |
| **METHODS** | | | |
| **Eligibility criteria** | 3 | Specify the inclusion and exclusion criteria for the review. | Yes |
| **Information sources** | 4 | Specify the information sources (e.g. databases, registers) used to identify studies and the date when each was last searched. | Yes |
| **Risk of bias** | 5 | Specify the methods used to assess risk of bias in the included studies. | Yes |
| **Synthesis of results** | 6 | Specify the methods used to present and synthesize results. | Yes |
| **RESULTS** | | | |
| **Included studies** | 7 | Give the total number of included studies and participants and summarize relevant characteristics of studies. | Yes |
| **Synthesis of results** | 8 | Present results for main outcomes, preferably indicating the number of included studies and participants for each. If meta-analysis was done, report the summary estimate and confidence/credible interval. If comparing groups, indicate the direction of the effect (i.e. which group is favored). | Yes |
| **DISCUSSION** | | | |
| **Limitations of evidence** | 9 | Provide a brief summary of the limitations of the evidence included in the review (e.g. study risk of bias, inconsistency and imprecision). | Yes |
| **Interpretation** | 10 | Provide a general interpretation of the results and important implications. | Yes |
| **OTHER** | | | |
| **Funding** | 11 | Specify the primary source of funding for the review. | No funding |
| **Registration** | 12 | Provide the register name and registration number. | Yes |

[a] Duplicate entries across PRISMA checklists were removed, but the original numbering was retained.

**Table 3**. PRISMA 2021 search extension checklist.

| Topic | No. | Item | Location reported (or details) |
|---|---|---|---|
| **INFORMATION SOURCES AND METHODS** | | | |
| **Objectives** | 1 | Name each individual database searched, stating the platform for each. | Appendix – Table 4 |
| **Database name** | 2 | If databases were searched simultaneously on a single platform, state the name of the platform, listing all of the databases searched. | Databases searched individually |
| **Study registries** | 3 | List any study registries searched. | Appendix – Table 4 |
| **Online resources and browsing** | 4 | Describe any online or print source purposefully searched or browsed (e.g., tables of contents, print conference proceedings, web sites), and how this was done. | None searched, besides preliminary scoping searches on Google Scholar. |
| **Citation searching** | 5 | Indicate whether cited references or citing references were examined, and describe any methods used for locating cited/citing references (e.g. browsing reference lists, using a citation index, setting up email alerts for references citing included studies). | Main text – Section 2.2 |
| **Contacts** | 6 | Indicate whether additional studies or data were sought by contacting authors, experts, manufacturers, or others. | Main text – Section 2.3, Appendix – Table 5 |
| **Other methods** | 7 | Describe any additional information sources or search methods used. | Main text – Figure 1 |
| **SEARCH STRATEGIES** | | | |
| Full search strategies | 8 | Include the search strategies for each database and information source, copied and pasted exactly as run. | Appendix – Table 4 |
| Limits and restrictions | 9 | Specify that no limits were used, or describe any limits or restrictions applied to a search (e.g. date or time period, language, study design) and provide justification for their use. | Main text – Section 2.2 |
| Search filters | 10 | Indicate whether published search filters were used (as originally designed or modified), and if so, cite the filter(s) used. | Appendix – Table 4 |
| Prior work | 11 | Indicate when search strategies from other literature reviews were adapted or reused for a substantive part or all of the search, citing the previous review(s). | Not used |
| Updates | 12 | Report the methods used to update the search(es) (e.g. rerunning searches, email alerts). | Search run only once |
| Dates of searches | 13 | For each search strategy, provide the date when the last search occurred. | Appendix – Table 4 |

| Topic | No. | Item | Location reported (or details) |
|---|---|---|---|
| **PEER REVIEW** | | | |
| Peer review | 14 | Describe any search peer review process. | No peer review |
| **MANAGING RECORDS** | | | |
| Total records | 15 | Document the total number of records identified from each database and other information sources. | Main text – Figure 1 |
| Deduplication | 16 | Describe the processes and any software used to deduplicate records from multiple database searches and other information sources. | Main text – Section 2.2 |

**Table 4.** Database searches.

| Database | Search date | Filters used | Search terms |
|---|---|---|---|
| MEDLINE (via PubMed) | 25/5/21 | Male<br>Adult: 19+ years | (hypogonadism OR testosterone OR cortisol OR sex hormone OR sex hormones OR androgen OR androgens) AND (low fat OR high fat OR high protein OR low protein OR high carbohydrate OR low carbohydrate OR ketogenic OR carbohydrate restricted OR carbohydrate limited OR dietary fat OR dietary carbohydrate OR dietary protein) AND (diet OR dietary) |
| CENTRAL | 25/5/21 | Trials<br>Search word variations | All text: (hypogonadism OR testosterone OR cortisol OR sex hormone OR sex hormones OR androgen OR androgens) AND (low fat OR high fat OR high protein OR low protein OR high carbohydrate OR low carbohydrate OR ketogenic OR carbohydrate restricted OR carbohydrate limited OR dietary fat OR dietary carbohydrate OR dietary protein) AND (diet OR dietary) |
| CINAHL Complete (via EBSCO) | 25/5/21 | Apply equivalent subjects<br>All adult<br>Male | (hypogonadism OR testosterone OR cortisol OR sex hormone OR sex hormones OR androgen OR androgens) AND (low fat OR high fat OR high protein OR low protein OR high carbohydrate OR low carbohydrate OR ketogenic OR carbohydrate restricted OR carbohydrate limited OR dietary fat OR dietary carbohydrate OR dietary protein) |
| SPORTDiscus with full text (via EBSCO) | 25/5/21 | Apply equivalent subjects | (hypogonadism OR testosterone OR cortisol OR sex hormone OR sex hormones OR androgen OR androgens) AND (low fat OR high fat OR high protein OR low protein OR high carbohydrate OR low carbohydrate OR ketogenic OR carbohydrate restricted OR carbohydrate limited OR dietary fat OR dietary carbohydrate OR dietary protein) |
| ClincalTrials.gov | 25/5/21 | Male<br>Adult (18-64)<br>Older Adult (65+)<br>Completed<br>Accepts healthy volunteers | Condition: low fat OR high fat OR high protein OR low protein OR high carbohydrate OR low carbohydrate OR ketogenic OR carbohydrate restricted OR carbohydrate limited OR dietary fat OR dietary carbohydrate OR dietary protein<br>Other terms: hypogonadism OR testosterone OR cortisol OR sex hormone OR sex hormones OR androgen OR androgens |
| ICTRP | 25/5/21 | With results only | hypogonadism OR testosterone OR cortisol OR sex hormone OR sex hormones OR androgen OR androgens AND low fat OR high fat OR high protein OR low protein OR high carbohydrate OR low carbohydrate OR ketogenic OR carbohydrate restricted OR carbohydrate limited OR dietary fat OR dietary carbohydrate OR dietary protein |

| Database | Search date | Filters used | Search terms |
| --- | --- | --- | --- |
| Open Grey | 25/5/21 | No filters | (hypogonadism OR testosterone OR cortisol OR sex hormone OR sex hormones OR androgen OR androgens) AND (diet OR dietary OR macronutrient OR macronutrients OR nutrition OR nutritional) |
| Google Scholar (search 1) | 26/5/21 | No filters | testosterone men "low carbohydrate" |
| Google Scholar (search 2) | 26/5/21 | No filters | cortisol men "low carbohydrate" |

**1. Additional considerations regarding data extraction**

**Krings 2021**

This study was originally a parallel study with one group receiving a low-carbohydrate (LC) diet, and the other group a LC diet plus carbohydrate supplements around workouts (Krings et al., 2021). The latter group was excluded in the data extraction and meta-analyses, as the carbohydrate supplementation made the group incomparable with the other studies. This in effect turned the study into a single-arm crossover study, and was analysed accordingly.

**Burke 2020**

This study was conducted over three separate time periods, using some of the same participants, which in effect made it both a crossover and parallel study. The study included two high-carbohydrate (HC) diet groups: a normal HC diet and a periodised HC diet (Burke et al., 2017, 2020). The latter group was excluded, as the periodisation of carbohydrate intake made it incomparable to the other included studies. This study used 13 of the same participants as Burke 2021 (Burke et al., 2021). To avoid double counting participants, these participants were excluded from the Burke 2020 data.

The authors of Burke 2020 supplied the individual participant data for both cortisol and testosterone, as this data is not yet published. Mean ± SD (change scores) were calculated for LC and HC diets using all of the participant observations provided.

Due to the complexity of this study, sample and diet characteristics data were drawn from the first two out of three trial periods only. This meant the data included five participants excluded, and excluded four participants included, in the meta-analyses. However, as all samples within this study were homogenous, the extracted data could be fairly generalized to the sample used in the meta-analyses.

**Bisschop 2000**

This study had two HC diets that were eligible (Bisschop et al., 2000). One of the HC diets had 0% fat intake, making it unsuitable for comparison with the other studies. Therefore, this diet was excluded in favour of the 'control' diet, which had 41% fat intake. The results of the two HC diets were not significantly different.

**Jaffe 2018**

The main report of this study was published in a possible predatory journal (Beall, 2021; Jaffe et al., 2018). The report itself was well written, and the study was methodologically sound. The lead author was contacted and confirmed the authenticity of the study. The author also replied that the study was done as his Master's thesis at Springfield College, USA (Jaffe, 2013). The thesis was independently sourced, which confirmed the authenticity of the study.

**Witt 1993**

This study had two subgroups, one that exercised during the dietary interventions and one that was sedentary (Witt et al., 1993). These subgroups were combined for the meta-analyses. Also, this study reported both day and night-time cortisol measurements. The daytime cortisol measurements were used for the 0hr post-exercise cortisol meta-analysis, as these were comparable to the other studies, which all reported daytime measurements.

The study had two eligible HC diets. The HC diet with the highest carbohydrate intake (75%) was used, to maximise the difference in carbohydrate intake between intervention diets. There was no significant difference in outcomes between the two HC diets.

**Tsai 1993**

This study had two eligible HC diets (Tsai et al., 1993). The intervention diets lacked a sufficient washout period. Therefore, the second HC diet ('carbohydrate rich diet ad libitum (CHO)') was excluded to provide an adequate washout period, for the majority of participants. The results of the two HC diets were not significantly different, however there was a non-significant increase in cortisol on the excluded HC diet.

**Galbo 1979**

This study included an exercise to exhaustion protocol, with blood samples taken every 30 min (Galbo et al., 1979). To standardize the exercise duration between LC and HC diets (LC, 61.5 min; HC, 106 min), 60 min cortisol values were extracted for both diets, for use in the meta-analyses. Also, this study had two identical LC diet periods. To maximise precision, a mean of these two diet periods was used.

**Table 5.** Excluded studies.

| Author & year | Reason for exclusion | Additional details |
|---|---|---|
| Anderson 1991 (Anderson et al., 1991; Thorp et al., 1990) | LC diet, carbohydrate intake >35% | The LC diet was <300 g/day carbohydrate and ~3000 kcal/day. Thus, using 299 g/day carbohydrate, carbohydrate intake was ~39.9%. |
| Ebbeling 2018 (Ebbeling et al., 2018, 2020; Ludwig, 2014) | Weight loss >3 kg | Before the HC and LC diets, the study included a 12% 'run-in' weight loss diet, making it too methodically heterogeneous to compare with the other included studies. |
| Helie 1985 (Hélie et al., 1985) | >15% difference in energy intake between HC & LC diets | Participants on the LC diet fasted before testing, whereas on the HC diet they ate breakfast. Since, the diets were one day long, this difference in food intake represented ~33% difference in energy intake. |
| Joy 2017 (Joy, 2017) | No outcome data | Based on the trial protocol this study appears eligible. However, to the best our knowledge the results are not yet publicly available. The author was emailed to request the results, but did not respond. |
| Kirwan 1990 (Kirwan et al., 1988, 1990) | LC diet, carbohydrate intake >35% | The LC diet was ~260 g/day carbohydrate. Energy intake was not reported, but the diets were 'eucaloric'. Using the available information and the Mifflin-St Jeor equation (Mifflin et al., 1990), multiplied by activity factor 1.725, energy intake was ~2812 kcal/day, making carbohydrate intake ~37%. |
| Reed 1987 (Reed et al., 1987) | LC diet, carbohydrate intake >35% | The LC diet was >100 g/day fat intake. Protein, carbohydrate, and energy intake were not reported. Protein intake was estimated to be 15.5%, based on a contemporary sample (Cohen et al., 2015). Energy intake was estimated to be 2500 kcal/day (Public Health England, 2016), as the participants were 97 ± 7% ideal bodyweight and the diets were 'weight-maintaining'. Using these figures and 101 g/day carbohydrate, carbohydrate intake was ~48.1%. |
| Remer 1998 (Remer et al., 1998; Remer and Manz, 1994) | LC diet, carbohydrate intake >35% | For the male participants, the LC diet was 2457 kcal/day and ~256 g/day carbohydrate, including ~30 g/day carbohydrate from supplements, making carbohydrate intake ~41.7%. |

| Author & year | Reason for exclusion | Additional details |
| --- | --- | --- |
| Sims 1974 (Sims et al., 1968, 1974) | No outcome data | This study reported measuring plasma and urinary cortisol. However, only the results for urinary cortisol were presented. Due to the age of the study, the authors were unable to be contacted to request the results. |
| Volek 2001 (Volek et al., 2000, 2001) | Weight loss >3 kg | Mean weight loss was 4.2 kg on the LC diet. |
| Wang 2005 (Wang et al., 2005) | LC diet, carbohydrate intake >35% | The LC diet was 37.9% fat. Carbohydrate and protein intake were not reported. Protein intake was estimated to be 15.6%, based on a contemporary sample (Cohen et al., 2015). Thus, carbohydrate intake was ~46.5%. |
| Wilson 2020 (Rauch et al., 2014; Silva, 2014; Wilson et al., 2020) | Carbohydrate loading on LC diet | The last week of the LC diet included carbohydrate loading. Outcome data before this point was not available. |

HC: high-carbohydrate; LC: low-carbohydrate.

**Table 6.** Characteristics of included studies.

| Author & year[a] | Study design | Sample size | Age | Body mass (kg) | BMI ($kg/m^2$) | Exercise during intervention[b] | Duration (min) x intensity (%$VO_{2max}$) of exercise before post-exercise cortisol[c] | Duration of LC diet (days) | Duration of HC diet (days) | Change in body mass during LC diet (kg) | Change in body mass during HC diet (kg) |
|---|---|---|---|---|---|---|---|---|---|---|---|
| Paoli 2021 (Paoli, 2020; Paoli et al., 2021) | Parallel | 19 | 27.4 ± 10.5 | 87.8 ± 13.3 | 26.8 ± 1.9 | Resistance | NA | 56 | 56 | -0.88 | 1.33 |
| Toma 2009 (Toma, 2009; Werner, 2006)[d] | Parallel | 17 | 23.3 ± 3.3 | 77.9 ± 11.4 | 24.2 | Aerobic | NA | 49 | 49 | -1.57 | -0.11 |
| Volek 2002 (Volek et al., 2002) | Parallel | 20 | 36 ± 11.9 | 81.7 ± 10.5 | ND | Mixed (n = 14) Aerobic (n = 5) Sedentary (n = 1) | NA | 42 | 42 | -2.2 | 0.4 |
| Shaw 2021 (Shaw et al., 2019, 2021) | Crossover (AB/BA) | 8 | 29.6 ± 5.1 | 73.1 ± 6.9 | 22.4 ± 1.7 | Aerobic | 232 x 70 (running) | 31 | 31 | -1.4 | -0.2 |
| Durkalec-Michalski 2021 (Durkalec-Michalski et al., 2019, 2021) | Crossover (AB) | 11 | 28.5 ± 5.3 | 81.3 ± 9.5 | 26 | Mixed | 18 x ~60-100 (cycling) | 28 | 14 (baseline diet) | -1.2 | 0 |

| Author & year[a] | Study design | Sample size | Age | Body mass (kg) | BMI ($kg/m^2$) | Exercise during intervention[b] | Duration (min) x intensity ($\%VO_{2max}$) of exercise before post-exercise cortisol[c] | Duration of LC diet (days) | Duration of HC diet (days) | Change in body mass during LC diet (kg) | Change in body mass during HC diet (kg) |
|---|---|---|---|---|---|---|---|---|---|---|---|
| Michalczyk 2019 (Michalczyk et al., 2019) | Crossover (AB) | 15 | 23.5 ± 2.2 | 92.2 ± 5.1 | 25 ± 1.9 | Mixed | NA | 28 | baseline diet | -1.8 | 0 |
| Waldman 2020 (Waldman et al., 2019, 2020) | Crossover (AB) | 15 | 33.5 ± 9.7 | 89.1 ± 12.7 | 28.1 ± 3.5 | Resistance (n = 14) Mixed (n = 1) | NA | 28 | 15 (baseline diet) | -2.64 | 0.1 |
| Zajac 2014 (Zajac et al., 2014) | Crossover (AB/BA) | 8 | 28.3 ± 3.9 | 80.3 ± 7.4 | 24.9 ± 3 | Aerobic | 105 x ~64-86 (cycling) | 28 | 28 | -2.08 | -0.2 |
| Krings 2021 (Krings, 2018; Krings et al., 2021) | Crossover (AB) | 9 | 19.7 ± 1 | 83.8 ± 12.6 | 26.1 | Mixed | NA | 28 | 14 (baseline diet) | 0.6 | 0 |
| Burke 2020 (Burke, 2019; Burke et al., 2017, 2020; Heikura et al., 2019; McKay, 2020; Mirtschin et al., 2018) | Parallel & crossover (A/B/AB) | 13 | 26.9 ± 3.9[e] | 65.3 ± 7.7[e] | ND | Aerobic | NA | 21 | 21 | -1.8[e] | -0.6[e] |

| Author & year[a] | Study design | Sample size | Age | Body mass (kg) | BMI (kg/m$^2$) | Exercise during intervention[b] | Duration (min) x intensity (%$VO_{2max}$) of exercise before post-exercise cortisol[c] | Duration of LC diet (days) | Duration of HC diet (days) | Change in body mass during LC diet (kg) | Change in body mass during HC diet (kg) |
|---|---|---|---|---|---|---|---|---|---|---|---|
| Gray 1990 (Gray et al., 1990)[d] | Crossover (AB) | 10 | 19-41 (range) | 80.3 ± 8.2 | ND | Mixed | NA | 18 | 12 | -1.9 | 0 |
| Waldman 2018 (Waldman et al., 2018) | Crossover (AB) | 11 | 21.6 ± 1.9 | 88.9 ± 11.3 | 27.6 | Mixed | NA | 15 | baseline diet | -1.2 | 0 |
| Terink 2021 (Mensink, 2019; Terink et al., 2021) | Crossover (AB/BA) | 14 | 32.9 ± 8.2 | 76.4 ± 5.4 | 23.1 ± 1.4 | Aerobic (n = 8) Mixed (n = 4) Resistance (n = 2) | 90 x 70 (cycling) | 14 | 14 | -2.4 | -1.3 |
| Bisschop 2000 (Bisschop et al., 2000) | Crossover (ABC/BAC/ CAB)[f] | 6 | 29-55 (range) | ND | 21-26 (range) | Sedentary | NA | 11 | 11 | ND | ND |
| Anderson 1987 (Alvares et al., 1976; Anderson et al., 1987) | Crossover (AB) | 7 | 22-43 (range) | 64-72 (range) | ND | Mixed | NA | 10 | 10 | ‘no significant changes’ | ‘no significant changes’ |
| Jaffe 2018 (Jaffe, 2013; Jaffe et al., 2018)[d] | Crossover (AB/BA) | 10 | 22.6 ± 4.1 | 81.3 ± 10.8 | 26.6 | Mixed | NA | 7 | 7 | -0.4 | -0.5 |

| Author & year[a] | Study design | Sample size | Age | Body mass (kg) | BMI (kg/m$^2$) | Exercise during intervention[b] | Duration (min) x intensity (%$VO_{2max}$) of exercise before post-exercise cortisol[c] | Duration of LC diet (days) | Duration of HC diet (days) | Change in body mass during LC diet (kg) | Change in body mass during HC diet (kg) |
|---|---|---|---|---|---|---|---|---|---|---|---|
| Burke 2021 (Burke, 2018; Burke et al., 2021; McKay, 2020; Mirtschin et al., 2018) | Parallel | 13 | 30.3 ± 4.3 | 66.2 ± 7.6 | ND | Aerobic | 125 x 75 (race walking) | 6 | 6 | -1.3 | -0.7 |
| Witt 1993 (Witt et al., 1993) | Crossover (ABC/BAC/ CAB/CBA)[g] | 15 | 23.1 ± 4 | 74.2 ± 7.6 | 24.8 | Aerobic (n = 8) Sedentary (n = 7) | NA | 5 | 5 | ND | ND |
| Lane 2010 (Duke Jr., 2008; Lane, 2009; Lane et al., 2010) | Parallel | 20 | 25.8 ± 5.1 | 74.9 ± 7.3 | 23.2 | Aerobic | 60 x 75 (cycling) | 4 | 4 | ND | ND |
| Galbo 1979 (Galbo et al., 1979) | Crossover (ABA)[h] | 7 | 26 | 77 | 22.7 | Aerobic | 60 x 70 (running) | 4 | 4 | -2 | -0.6 |
| Gleeson 1998 (Gleeson et al., 1998) | Parallel | 12 | 25 ± 6.9 | 78.3 ± 11.8 | 24.4 | Aerobic | 60 x 70 (cycling) | 3 | 3 | ND | ND |
| Bishop 2001 (Bishop et al., 2001a, 2001b) | Crossover (AB/BA) | 12 | 26 ± 6.9 | 73.4 ± 9 | 23.2 | Aerobic | 90 x ~70-93 (cycling) | 3 | 3 | -0.5 | 0.8 |

| Author & year[a] | Study design | Sample size | Age | Body mass (kg) | BMI (kg/m$^2$) | Exercise during intervention[b] | Duration (min) x intensity (%$VO_{2max}$) of exercise before post-exercise cortisol[c] | Duration of LC diet (days) | Duration of HC diet (days) | Change in body mass during LC diet (kg) | Change in body mass during HC diet (kg) |
|---|---|---|---|---|---|---|---|---|---|---|---|
| Tsai 1993 (Tsai et al., 1993) | Crossover (ABC/ACB)[i] | 4 | 25.5 ± 1.7 | 78.3 ± 3.3 | 23.6 ± 1.3 | Mixed | NA | 3 | baseline diet | no significant changes | no significant changes |
| Langfort 1996 (Langfort et al., 1994, 1996) | Crossover (AB) | 8 | 22 ± 0.9 | 69 ± 9 | 23.1 | Aerobic | 27 x ~60-100 (cycling) | 3 | 3 | ND | ND |
| Langfort 2001 (Langfort et al., 2001) | Crossover (AB) | 9 | 22 ± 0.9 | 77.1 ± 2.8 | 24.6 | Aerobic | NA | 3 | 3 | ND | ND |
| Lima-Silva 2011 (Lima-Silva et al., 2011) | Crossover (AB/BA) | 6 | 25.8 ± 5.8 | 70.9 ± 6.3 | 22.8 | Aerobic | 15 x ~60-90 (cycling) | 2 | 2 | ND | ND |
| Mitchell 1998 (Mitchell et al., 1998) | Crossover (AB/BA) | 10 | 24.7 ± 15.2 | 79.32 ± 23.6 | 24.8 | Aerobic | 60 x 75 (cycling) | 2 | 2 | -0.85 | 0.08 |

[a] Unless otherwise stated, all data pertains to the participants and interventions included in the meta-analyses.
[b] Aerobic (≥80% aerobic training), resistance (≥80% resistance training), mixed (combination of aerobic, resistance, sport, or unspecified exercise).
[c] All exercise was aerobic. x - x indicates increasing intensity during exercise. Where $VO_{2max}$ was not provided, it was estimated from the available data and indicated using ~.
[d] Studies from grey literature.
[e] Data includes five participants excluded, and excludes for participants included, in the meta-analyses.
[f] Two HC diets eligible. Data extracted for 'control' diet.
[g] Two HC diets eligible. Data extracted for 'high carbohydrate (low fat) diet'.

[h] Two LC diets eligible. Data extracted both LC diets, and a mean presented.
[i] Two HC diets eligible. Data extracted for ‘western type food (N)’ diet.
Values presented as mean ± SD.
BMI: body mass index; HC: high-carbohydrate; LC: low-carbohydrate; NA: not applicable; ND: no data; SD: standard deviation; $VO_{2max}$: maximal oxygen uptake.

**Table 7.** Characteristics of study diets.

| Study[a] | LC diet | | | | | | | HC diet | | | | | | |
|---|---|---|---|---|---|---|---|---|---|---|---|---|---|---|
| | Carbo-hydrate (%) | Fat (%) | Protein (%) | Fiber (g) | Sugar (g) | Cholesterol (mg) | Energy (kcal) | Carbo-hydrate (%) | Fat (%) | Protein (%) | Fiber (g) | Sugar (g) | Cholesterol (mg) | Energy (kcal) |
| Paoli 2021 | 5 | 68 | 24.7 | ND | ND | ND | 3443.7 | 55 | 20 | 25 | ND | ND | ND | 3529.7 |
| Toma 2009 | 30.6 | 38.5 | 29.9 | ND | ND | ND | 2605.5 | 57.5 | 25.4 | 14.2 | ND | ND | ND | 2540.5 |
| Volek 2002 | 8 | 61 | 30 | ND | ND | 741 | 2333.5 | 58 | 26 | 16 | ND | ND | 155 | 1949 |
| Shaw 2021[b] | 4.1 | 77.7 | 18.2 | 23.3 | 24 | ND | 3279.4 | 42.9 | 38.5 | 18.6 | 41.1 | 128 | ND | 3121.7 |
| Durkalec-Michalski 2021[b] | 4 | 77 | 18 | 8.9 | 2 | 637 | 2955 | 44 | 36 | 19 | 34.7 | 37.3 | 497 | 2645 |
| Michalczyk 2019 | 10 | 59 | 31 | ND | ND | ND | 3758 | 54 | 31 | 15 | ND | ND | ND | 3740 |
| Waldman 2020 | 21 | 48.5 | 30.5 | 15.3 | 30 | 221.3 | 1759 | 47 | 35 | 18 | 14 | 87 | 323 | 2090 |
| Zajac 2014 | 15 | 70 | 15 | ND | ND | ND | 3865 | 50 | 30 | 20 | ND | ND | ND | 3865 |
| Krings 2021 | 22.7 | 51.3 | 26 | ND | ND | ND | 2944.6 | 44.2 | 32.4 | 22.4 | ND | ND | ND | 2637.9 |
| Burke 2020[bc] | 3.8 | 78.8 | 16.1 | ND | 25 | 931 | 3472.8 | 62.4 | 19.7 | 15.7 | ND | 278 | 331 | 3518.2 |
| Gray 1990 (ranges shown) | 7 - 9 | 73 - 76 | 16 - 20 | ND | ND | ND | 3004 | 48 - 53 | 35 - 36 | 12 - 16 | ND | ND | ND | 3054 |
| Waldman 2018 | 14.3 | 58 | 28 | 13 | 30 | ND | 2457.7 | 52 | 29 | 19 | 14 | 76 | ND | 2357 |

| Study[a] | LC diet | | | | | | | HC diet | | | | | | |
|---|---|---|---|---|---|---|---|---|---|---|---|---|---|---|
| | Carbo-hydrate (%) | Fat (%) | Protein (%) | Fiber (g) | Sugar (g) | Cholesterol (mg) | Energy (kcal) | Carbo-hydrate (%) | Fat (%) | Protein (%) | Fiber (g) | Sugar (g) | Cholesterol (mg) | Energy (kcal) |
| Terink 2021 | 8 | 73 | 16 | 28 | ND | 699 | 3104 | 49 | 33 | 15 | 41 | ND | 165 | 3075 |
| Bisschop 2000[d] | 2 | 83 | 15 | ND | ND | ND | ND | 44 | 41 | 15 | ND | ND | ND | ND |
| Anderson 1987 | 35 | 21 | 44 | ND | ND | ND | ND | 70 | 20 | 10 | ND | ND | ND | ND |
| Jaffe 2018 | 7 | 39 | 55 | ND | ND | ND | 2271.2 | 55 | 20 | 25 | ND | ND | ND | 2294 |
| Burke 2021 | 4 | 80 | 16 | ND | ND | ND | 3530 | 66 | 20 | 15 | ND | ND | ND | 3852 |
| Witt 1993 | 15 | 70 | 15 | ND | ND | ND | ND | 75 | 10 | 15 | ND | ND | ND | ND |
| Lane 2010 | 31.9 | ND | ND | ND | ND | ND | 2804.1 | 58.5 | ND | ND | ND | ND | ND | 3226.7 |
| Galbo 1979[d] | 10.5 | 76 | 13.5 | ND | ND | ND | 2641 | 77 | 9.5 | 13.5 | ND | ND | ND | 2904 |
| Gleeson 1998 | 7 | 68 | 25 | ND | ND | ND | 2221.3 | 75 | 17 | 8 | ND | ND | ND | 2006.3 |
| Bishop 2001 | 8.7 | 68.5 | 22.3 | ND | ND | ND | 3343.8 | 76.7 | 10.2 | 12.2 | ND | ND | ND | 2913.9 |
| Tsai 1993 | 15 | 60 | 25 | ND | ND | ND | 2500 - 3000 (range) | 45 | 40 | 15 | ND | ND | ND | 2500 - 3000 (range) |
| Langfort 1996 | 5 | 50 | 45 | ND | ND | ND | 2208 | 50 | 30 | 20 | ND | ND | ND | 2208 |

| Study[a] | LC diet | | | | | | | HC diet | | | | | | |
|---|---|---|---|---|---|---|---|---|---|---|---|---|---|---|
| | Carbo-hydrate (%) | Fat (%) | Protein (%) | Fiber (g) | Sugar (g) | Cholesterol (mg) | Energy (kcal) | Carbo-hydrate (%) | Fat (%) | Protein (%) | Fiber (g) | Sugar (g) | Cholesterol (mg) | Energy (kcal) |
| Langfort 2001 | 5 | 50 | 45 | ND | ND | ND | 2395.6 | 50 | 30 | 20 | ND | ND | ND | 2395.6 |
| Lima-Silva 2011 | 10 | 35 | 55 | ND | ND | ND | ND | 80 | 10 | 10 | ND | ND | ND | ND |
| Mitchell 1998 | 4.4 | 66.9 | 27.8 | ND | ND | ND | 3080 | 79.1 | 9.7 | 12.9 | ND | ND | ND | 3056 |

[a] Unless otherwise stated, all data pertains to the participants and interventions included in the meta-analyses.
[b] Studies reported data on micronutrient intakes.
[c] Data includes five participants excluded, and excludes four participants included, in the meta-analyses.
[d] Studies used liquid intervention diets.
Values presented as mean.
HC: high-carbohydrate; LC: low-carbohydrate; ND: no data.

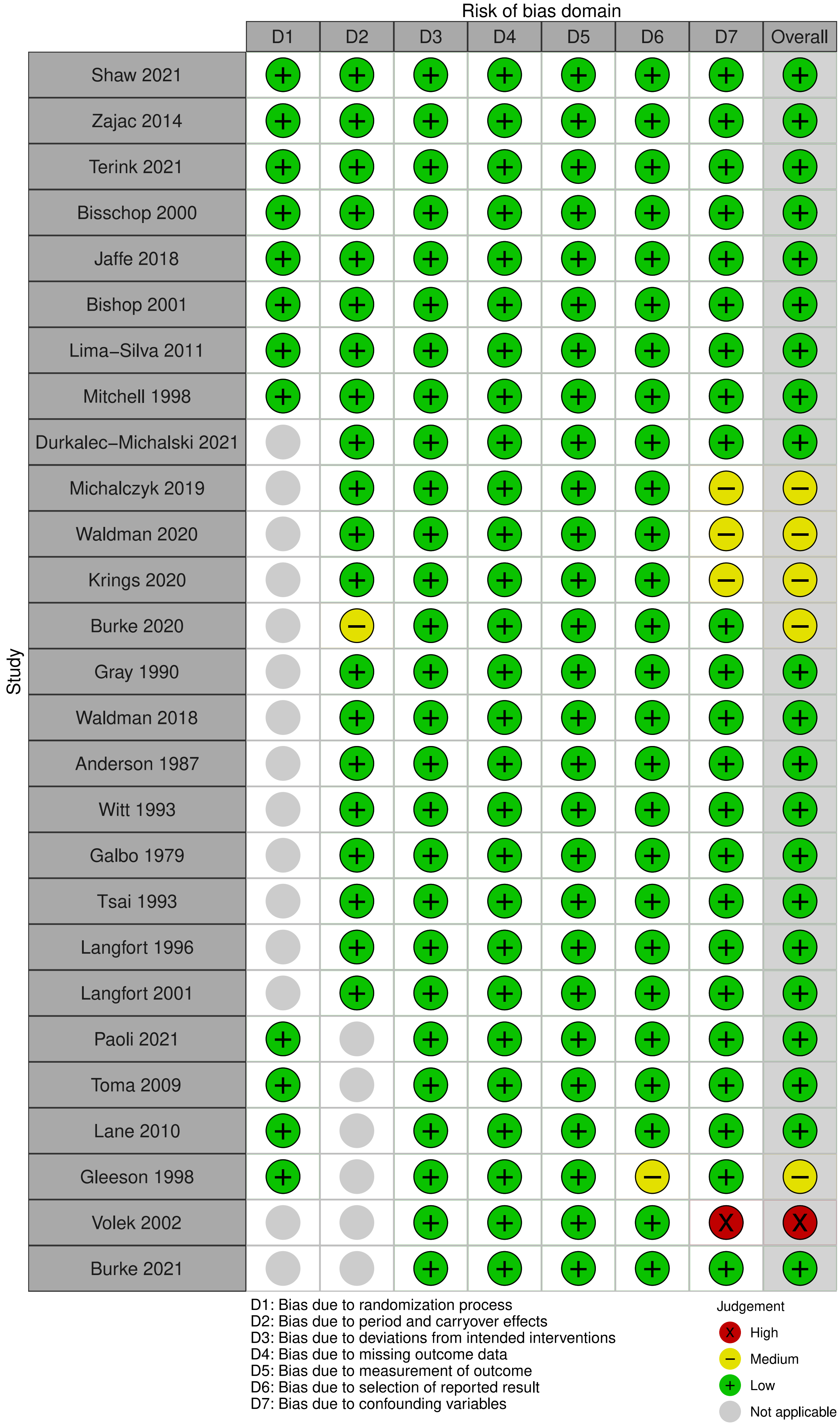


**Figure 1.** Risk of bias ratings for studies.

**Table 8.** Reasons for high and medium bias ratings.

| Study | Bias domain | Bias rating | Reason |
|---|---|---|---|
| Michalczyk 2019 | Bias due to confounding variables | Medium | During the LC diet period, the participants lived in a dormitory which may have altered their sleep habits, psychological stress levels, and social activity; all of which are possible confounding variables. The participants most likely did not live in a dormitory during the HC diet period, since this was their habitual diet. The author was contacted to clarify this point, but did not respond. |
| Waldman 2020 | Bias due to confounding variables | Medium | The LC vs HC diet was 15.8% lower in energy intake, and the difference in weight change was -2.74 kg. These were the highest differences in energy intake and weight change of any included study, and somewhat an outlier. The weight loss and reduced energy intake on the LC diet may have confounded the results. |
| Krings 2020 | Bias due to confounding variables | Medium | During the intervention diets resistance training volume was matched, whereas interval training volume was higher on the LC diet. This small difference in exercise volume may have confounded the results. |
| Burke 2020 | Bias due to period and carryover effects | Medium | The participant allocation between diets and time periods was not balanced, which put the results at risk of period effects. |
| Gleeson 1998 | Bias due to selection of reported result | Medium | This parallel study presented baseline data as combined (n = 12), but post-intervention data as split (n = 6, x2). Presenting the data in this way made it impossible to extract change from baseline scores, even though this data was available to the study's authors. This was most likely an honest error, but still raises some concerns over selective reporting. The lead author was contacted to request change from baseline data, but did not respond. |
| Volek 2002 | Bias due to confounding variables | High | The LC diet group received a daily multi-vitamin and mineral supplement, whereas the HC diet group did not. This difference in micronutrient supplementation is likely to have confounded the results. |

HC: high-carbohydrate; LC: low-carbohydrate.

## A. Cortisol, resting, long- vs short-term LC diets.

| Study or Subgroup | LC diet Mean | LC diet SD | LC diet Total | HC diet Mean | HC diet SD | HC diet Total | Weight | Std. Mean Difference IV, Random, 95% CI |
|---|---|---|---|---|---|---|---|---|
| **1.1.1 Long-term LC diets (≥3 weeks)** | | | | | | | | |
| Volek 2002 | -103 | 247 | 12 | 50 | 98 | 8 | 4.7% | -0.72 [-1.65, 0.21] |
| Shaw 2021 | 24 | 81.4 | 8 | -38 | 127 | 8 | 4.2% | 0.55 [-0.45, 1.55] |
| Durkalec-Michalski 2021 | 285.7 | 84.7 | 11 | 290.4 | 56.8 | 11 | 5.4% | -0.06 [-0.90, 0.77] |
| Michalczyk 2019 | 16.22 | 6.4 | 15 | 16.38 | 6.81 | 15 | 6.5% | -0.02 [-0.74, 0.69] |
| Waldman 2020 | 23.8 | 5 | 15 | 24.5 | 4.2 | 15 | 6.5% | -0.15 [-0.86, 0.57] |
| Zajac 2014 | 553 | 49 | 8 | 649 | 62 | 8 | 3.3% | -1.62 [-2.80, -0.45] |
| Burke 2020 | 35.23857 | 101.6751 | 7 | 73.48111 | 90.40971 | 8 | 4.1% | -0.38 [-1.40, 0.65] |
| **Subtotal (95% CI)** | | | **76** | | | **73** | **34.7%** | **-0.28 [-0.70, 0.15]** |
| Heterogeneity: Tau² = 0.12; Chi² = 9.40, df = 6 (P = 0.15); I² = 36% | | | | | | | | |
| Test for overall effect: Z = 1.28 (P = 0.20) | | | | | | | | |
| **1.1.2 Short-term LC diets (<3 weeks)** | | | | | | | | |
| Gray 1990 | 105.1 | 36.8 | 10 | 91.4 | 27.7 | 10 | 5.0% | 0.40 [-0.48, 1.29] |
| Waldman 2018 | 253.9 | 46.4 | 11 | 224.7 | 44.5 | 11 | 5.2% | 0.62 [-0.24, 1.48] |
| Terink 2021 | 510.3 | 229.7378 | 14 | 465.7 | 242.8336 | 14 | 6.2% | 0.18 [-0.56, 0.93] |
| Bisschop 2000 | 265 | 63.68673 | 6 | 217 | 51.43928 | 6 | 3.3% | 0.77 [-0.43, 1.96] |
| Anderson 1987 | 10.6 | 1.058301 | 7 | 7.74 | 1.878483 | 7 | 2.9% | 1.76 [0.46, 3.05] |
| Burke 2021 | 0.46 | 3.3 | 7 | -0.07 | 2.26 | 6 | 3.7% | 0.17 [-0.92, 1.26] |
| Witt 1993 | 8.933 | 2.5799 | 15 | 8.34 | 2.3427 | 15 | 6.5% | 0.23 [-0.48, 0.95] |
| Lane 2010 | 27.64 | 7.91 | 8 | 19.4 | 4.4 | 12 | 4.2% | 1.31 [0.31, 2.32] |
| Galbo 1979 | 21.6 | 100.0094 | 7 | -14.4 | 120.6463 | 7 | 3.9% | 0.30 [-0.75, 1.36] |
| Gleeson 1998 | 460 | 293.9388 | 6 | 615 | 247.3985 | 6 | 3.4% | -0.53 [-1.69, 0.63] |
| Bishop 2001 | 398 | 114 | 12 | 376 | 99 | 12 | 5.7% | 0.20 [-0.60, 1.00] |
| Tsai 1993 | 458 | 255 | 4 | 443 | 114 | 4 | 2.6% | 0.07 [-1.32, 1.45] |
| Langfort 1996 | 233.63 | 107.8479 | 8 | 190.11 | 113.4765 | 8 | 4.3% | 0.37 [-0.62, 1.36] |
| Lima-Silva 2011 | 497.5 | 307.4 | 6 | 431.4 | 97.9 | 6 | 3.5% | 0.27 [-0.87, 1.41] |
| Mitchell 1998 | 42.1 | 211.5564 | 10 | -75.3 | 213.1375 | 10 | 4.9% | 0.53 [-0.37, 1.42] |
| **Subtotal (95% CI)** | | | **131** | | | **134** | **65.3%** | **0.41 [0.16, 0.66]** |
| Heterogeneity: Tau² = 0.00; Chi² = 11.75, df = 14 (P = 0.63); I² = 0% | | | | | | | | |
| Test for overall effect: Z = 3.22 (P = 0.001) | | | | | | | | |
| **Total (95% CI)** | | | **207** | | | **207** | **100.0%** | **0.18 [-0.06, 0.42]** |
| Heterogeneity: Tau² = 0.11; Chi² = 30.77, df = 21 (P = 0.08); I² = 32% | | | | | | | | |
| Test for overall effect: Z = 1.44 (P = 0.15) | | | | | | | | |
| Test for subgroup differences: Chi² = 7.51, df = 1 (P = 0.006), I² = 86.7% | | | | | | | | |

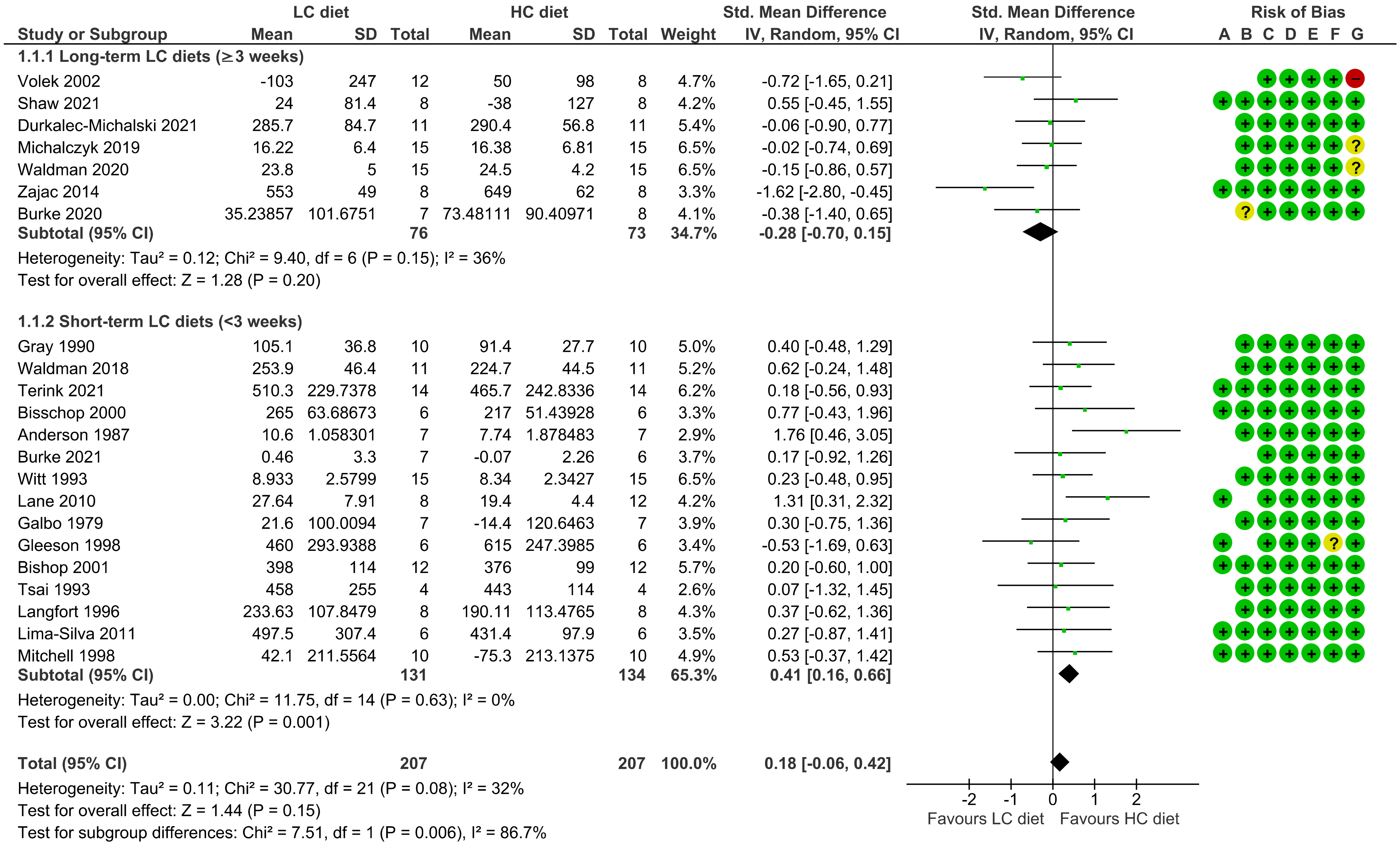

## B. Cortisol, 0 hr post-exercise, long- vs short-term LC diets.

| Study or Subgroup | LC diet Mean | LC diet SD | LC diet Total | HC diet Mean | HC diet SD | HC diet Total | Weight | Std. Mean Difference IV, Random, 95% CI |
|---|---|---|---|---|---|---|---|---|
| **1.2.1 Long-term LC diets (≥3 weeks)** | | | | | | | | |
| Shaw 2021 | 188 | 175 | 8 | -7 | 190 | 8 | 8.1% | 1.01 [-0.05, 2.07] |
| Durkalec-Michalski 2021 | 303.3 | 80.2 | 11 | 368.1 | 123.7 | 11 | 9.8% | -0.60 [-1.46, 0.26] |
| Zajac 2014 | 676 | 65 | 8 | 650 | 62 | 8 | 8.6% | 0.39 [-0.61, 1.38] |
| **Subtotal (95% CI)** | | | **27** | | | **27** | **26.5%** | **0.23 [-0.71, 1.17]** |
| Heterogeneity: Tau² = 0.45; Chi² = 5.66, df = 2 (P = 0.06); I² = 65% | | | | | | | | |
| Test for overall effect: Z = 0.47 (P = 0.64) | | | | | | | | |
| **1.2.2 Short-term LC diets (<3 weeks)** | | | | | | | | |
| Terink 2021 | 669 | 243 | 14 | 555 | 173 | 14 | 10.9% | 0.52 [-0.23, 1.28] |
| Burke 2021 | 12.75 | 3.16 | 7 | 0.28 | 4.71 | 6 | 4.3% | 2.94 [1.20, 4.68] |
| Lane 2010 | 27.89 | 6.75 | 8 | 22.47 | 8.25 | 12 | 9.2% | 0.67 [-0.25, 1.60] |
| Galbo 1979 | 752 | 190.4941 | 7 | 500.6 | 188.9066 | 7 | 7.2% | 1.24 [0.06, 2.42] |
| Gleeson 1998 | 793 | 578.0796 | 6 | 501 | 93.08061 | 6 | 7.2% | 0.65 [-0.52, 1.83] |
| Bishop 2001 | 711 | 203 | 12 | 560 | 81 | 12 | 9.9% | 0.94 [0.09, 1.80] |
| Langfort 1996 | 246.09 | 102.6153 | 8 | 159.56 | 152.1977 | 8 | 8.4% | 0.63 [-0.38, 1.64] |
| Lima-Silva 2011 | 354.4 | 151.5 | 6 | 519.4 | 218.1 | 6 | 7.0% | -0.81 [-2.01, 0.39] |
| Mitchell 1998 | 793.8 | 255.8283 | 10 | 638.8 | 255.8283 | 10 | 9.4% | 0.58 [-0.32, 1.48] |
| **Subtotal (95% CI)** | | | **78** | | | **81** | **73.5%** | **0.71 [0.26, 1.16]** |
| Heterogeneity: Tau² = 0.20; Chi² = 13.90, df = 8 (P = 0.08); I² = 42% | | | | | | | | |
| Test for overall effect: Z = 3.07 (P = 0.002) | | | | | | | | |
| **Total (95% CI)** | | | **105** | | | **108** | **100.0%** | **0.58 [0.17, 0.99]** |
| Heterogeneity: Tau² = 0.26; Chi² = 22.18, df = 11 (P = 0.02); I² = 50% | | | | | | | | |
| Test for overall effect: Z = 2.75 (P = 0.006) | | | | | | | | |
| Test for subgroup differences: Chi² = 0.82, df = 1 (P = 0.37), I² = 0% | | | | | | | | |

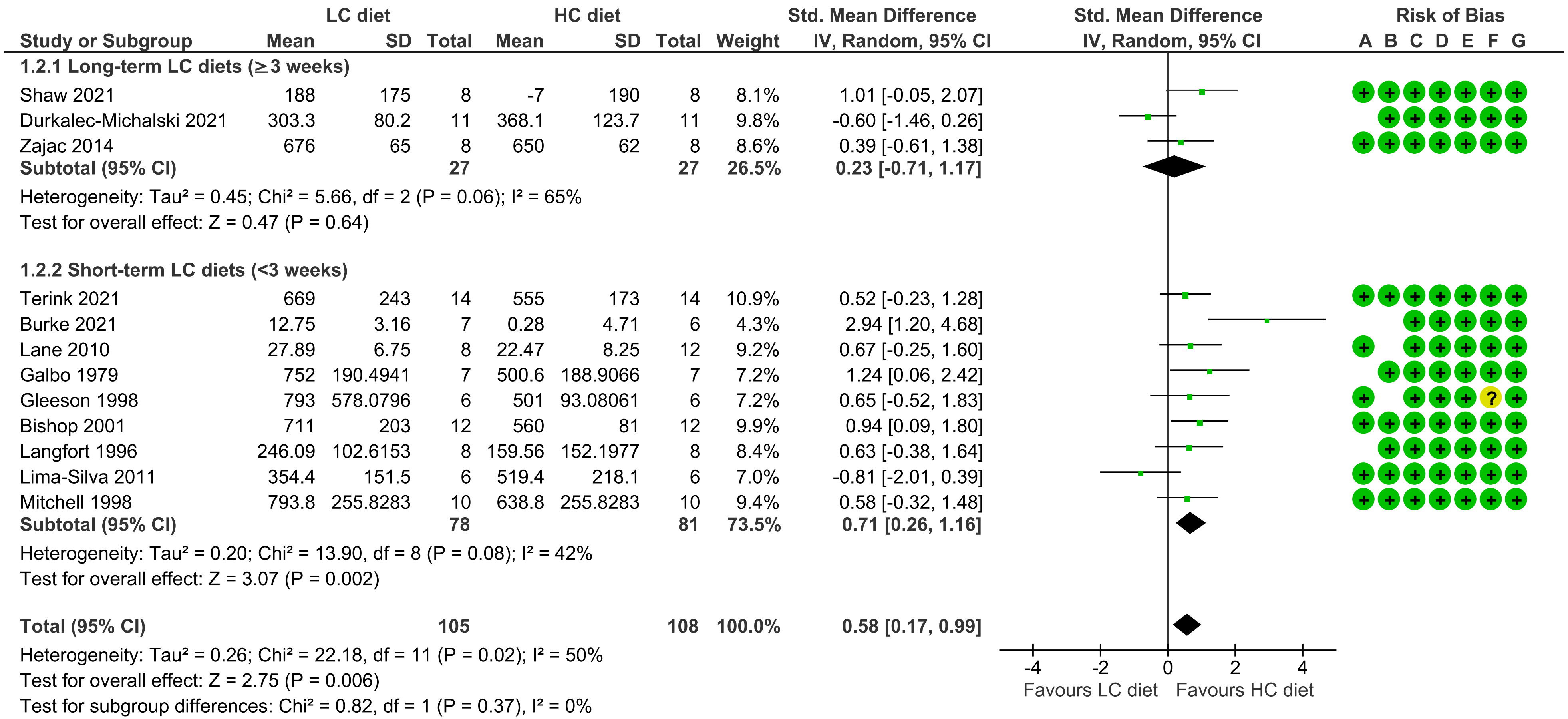

## C. Cortisol, 0 hr post-exercise, MP- vs HP-LC diets.

| Study or Subgroup | LC diet Mean | LC diet SD | LC diet Total | HC diet Mean | HC diet SD | HC diet Total | Weight | Std. Mean Difference IV, Random, 95% CI |
|---|---|---|---|---|---|---|---|---|
| **1.3.1 MP-LC diets (<35%)** | | | | | | | | |
| Shaw 2021 | 188 | 175 | 8 | -7 | 190 | 8 | 8.1% | 1.01 [-0.05, 2.07] |
| Durkalec-Michalski 2021 | 303.3 | 80.2 | 11 | 368.1 | 123.7 | 11 | 9.8% | -0.60 [-1.46, 0.26] |
| Zajac 2014 | 676 | 65 | 8 | 650 | 62 | 8 | 8.6% | 0.39 [-0.61, 1.38] |
| Terink 2021 | 669 | 243 | 14 | 555 | 173 | 14 | 10.9% | 0.52 [-0.23, 1.28] |
| Burke 2021 | 12.75 | 3.16 | 7 | 0.28 | 4.71 | 6 | 4.3% | 2.94 [1.20, 4.68] |
| Lane 2010 | 27.89 | 6.75 | 8 | 22.47 | 8.25 | 12 | 9.2% | 0.67 [-0.25, 1.60] |
| Galbo 1979 | 752 | 190.4941 | 7 | 500.6 | 188.9066 | 7 | 7.2% | 1.24 [0.06, 2.42] |
| Gleeson 1998 | 793 | 578.0796 | 6 | 501 | 93.08061 | 6 | 7.2% | 0.65 [-0.52, 1.83] |
| Bishop 2001 | 711 | 203 | 12 | 560 | 81 | 12 | 9.9% | 0.94 [0.09, 1.80] |
| Mitchell 1998 | 793.8 | 255.8283 | 10 | 638.8 | 255.8283 | 10 | 9.4% | 0.58 [-0.32, 1.48] |
| **Subtotal (95% CI)** | | | **91** | | | **94** | **84.5%** | **0.68 [0.25, 1.12]** |
| Heterogeneity: Tau² = 0.22; Chi² = 17.02, df = 9 (P = 0.05); I² = 47% | | | | | | | | |
| Test for overall effect: Z = 3.10 (P = 0.002) | | | | | | | | |
| **1.3.2 HP-LC diets (≥35%)** | | | | | | | | |
| Langfort 1996 | 246.09 | 102.6153 | 8 | 159.56 | 152.1977 | 8 | 8.4% | 0.63 [-0.38, 1.64] |
| Lima-Silva 2011 | 354.4 | 151.5 | 6 | 519.4 | 218.1 | 6 | 7.0% | -0.81 [-2.01, 0.39] |
| **Subtotal (95% CI)** | | | **14** | | | **14** | **15.5%** | **-0.05 [-1.46, 1.36]** |
| Heterogeneity: Tau² = 0.72; Chi² = 3.24, df = 1 (P = 0.07); I² = 69% | | | | | | | | |
| Test for overall effect: Z = 0.07 (P = 0.94) | | | | | | | | |
| **Total (95% CI)** | | | **105** | | | **108** | **100.0%** | **0.58 [0.17, 0.99]** |
| Heterogeneity: Tau² = 0.26; Chi² = 22.18, df = 11 (P = 0.02); I² = 50% | | | | | | | | |
| Test for overall effect: Z = 2.75 (P = 0.006) | | | | | | | | |
| Test for subgroup differences: Chi² = 0.96, df = 1 (P = 0.33), I² = 0% | | | | | | | | |

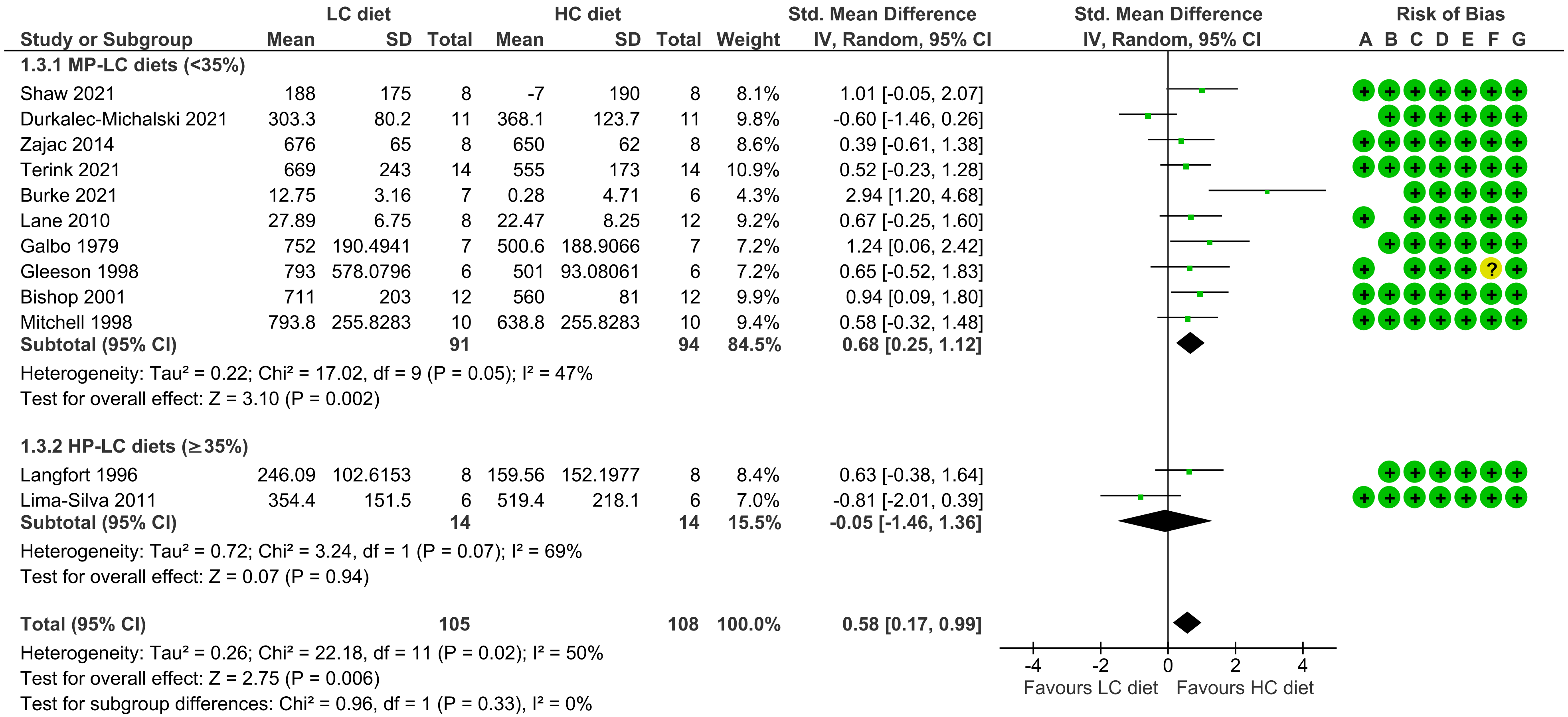

## D. Cortisol, 0 hr post-exercise, long- vs short-duration exercise.

| Study or Subgroup | LC diet Mean | LC diet SD | LC diet Total | HC diet Mean | HC diet SD | HC diet Total | Weight | Std. Mean Difference IV, Random, 95% CI |
|---|---|---|---|---|---|---|---|---|
| **1.4.1 Long-duration exercise (≥20min)** | | | | | | | | |
| Shaw 2021 | 188 | 175 | 8 | -7 | 190 | 8 | 8.1% | 1.01 [-0.05, 2.07] |
| Burke 2021 | 12.75 | 3.16 | 7 | 0.28 | 4.71 | 6 | 4.3% | 2.94 [1.20, 4.68] |
| Zajac 2014 | 676 | 65 | 8 | 650 | 62 | 8 | 8.6% | 0.39 [-0.61, 1.38] |
| Bishop 2001 | 711 | 203 | 12 | 560 | 81 | 12 | 9.9% | 0.94 [0.09, 1.80] |
| Terink 2021 | 669 | 243 | 14 | 555 | 173 | 14 | 10.9% | 0.52 [-0.23, 1.28] |
| Lane 2010 | 27.89 | 6.75 | 8 | 22.47 | 8.25 | 12 | 9.2% | 0.67 [-0.25, 1.60] |
| Mitchell 1998 | 793.8 | 255.8283 | 10 | 638.8 | 255.8283 | 10 | 9.4% | 0.58 [-0.32, 1.48] |
| Galbo 1979 | 752 | 190.4941 | 7 | 500.6 | 188.9066 | 7 | 7.2% | 1.24 [0.06, 2.42] |
| Gleeson 1998 | 793 | 578.0796 | 6 | 501 | 93.08061 | 6 | 7.2% | 0.65 [-0.52, 1.83] |
| Langfort 1996 | 246.09 | 102.6153 | 8 | 159.56 | 152.1977 | 8 | 8.4% | 0.63 [-0.38, 1.64] |
| **Subtotal (95% CI)** | | | **88** | | | **91** | **83.1%** | **0.78 [0.47, 1.10]** |
| Heterogeneity: Tau² = 0.00; Chi² = 8.26, df = 9 (P = 0.51); I² = 0% | | | | | | | | |
| Test for overall effect: Z = 4.89 (P < 0.00001) | | | | | | | | |
| **1.4.2 Short-duration exercise (<20min)** | | | | | | | | |
| Durkalec-Michalski 2021 | 303.3 | 80.2 | 11 | 368.1 | 123.7 | 11 | 9.8% | -0.60 [-1.46, 0.26] |
| Lima-Silva 2011 | 354.4 | 151.5 | 6 | 519.4 | 218.1 | 6 | 7.0% | -0.81 [-2.01, 0.39] |
| **Subtotal (95% CI)** | | | **17** | | | **17** | **16.9%** | **-0.67 [-1.37, 0.03]** |
| Heterogeneity: Tau² = 0.00; Chi² = 0.08, df = 1 (P = 0.78); I² = 0% | | | | | | | | |
| Test for overall effect: Z = 1.88 (P = 0.06) | | | | | | | | |
| **Total (95% CI)** | | | **105** | | | **108** | **100.0%** | **0.58 [0.17, 0.99]** |
| Heterogeneity: Tau² = 0.26; Chi² = 22.18, df = 11 (P = 0.02); I² = 50% | | | | | | | | |
| Test for overall effect: Z = 2.75 (P = 0.006) | | | | | | | | |
| Test for subgroup differences: Chi² = 13.84, df = 1 (P = 0.0002), I² = 92.8% | | | | | | | | |

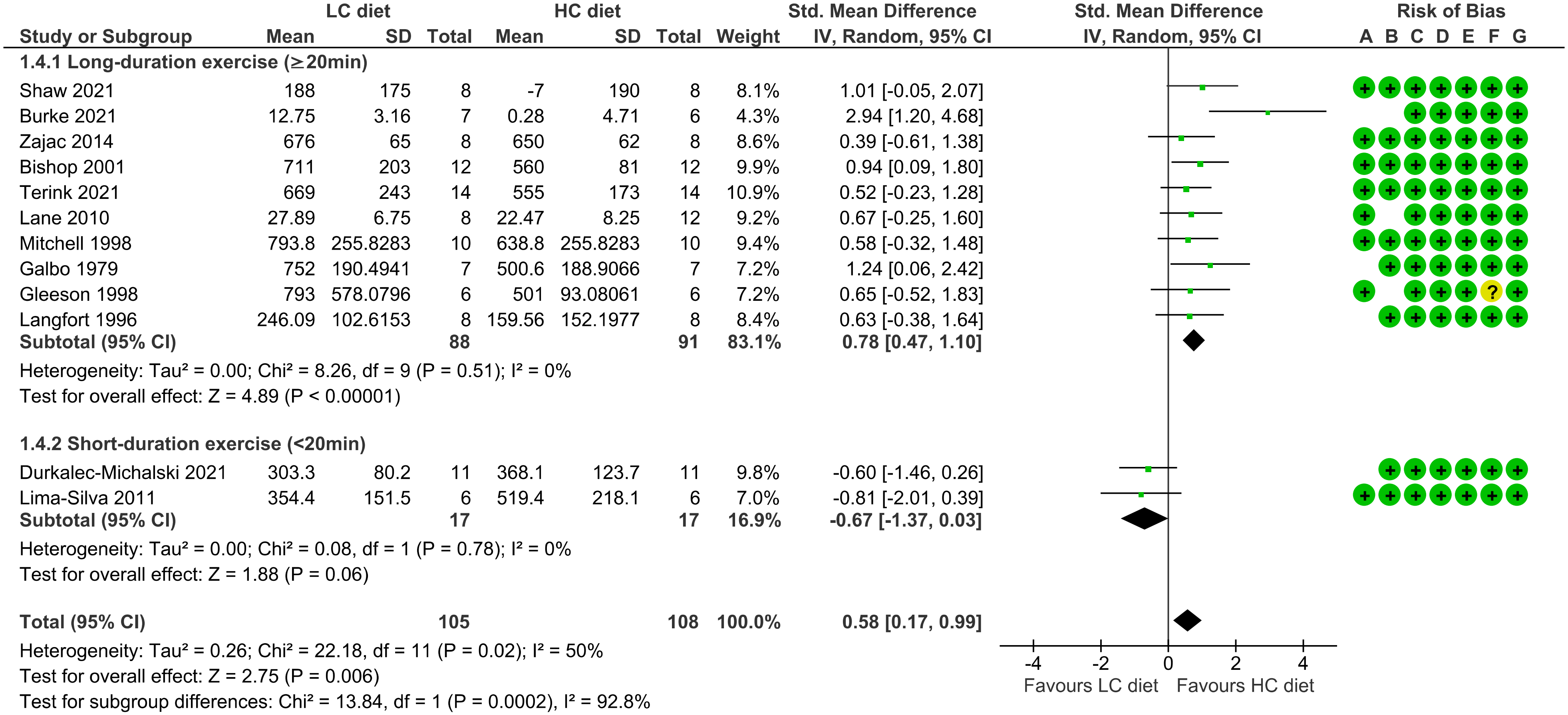

**E. Cortisol, 1 hr post-exercise (all studies: long-duration exercise only).**

| Study or Subgroup | LC diet Mean | LC diet SD | LC diet Total | HC diet Mean | HC diet SD | HC diet Total | Weight | Std. Mean Difference IV, Random, 95% CI | Risk of Bias A | B | C | D | E | F | G |
|---|---|---|---|---|---|---|---|---|---|---|---|---|---|---|---|
| Shaw 2021 | 132 | 176 | 8 | -20 | 153 | 8 | 18.0% | 0.87 [-0.17, 1.91] | + | + | + | + | + | + | + |
| Terink 2021 | 516.8 | 225.2478 | 14 | 418.2 | 239.8402 | 14 | 28.8% | 0.41 [-0.34, 1.16] | + | + | + | + | + | + | + |
| Burke 2021 | 11.45 | 5.66 | 7 | -0.03 | 3.87 | 6 | 10.0% | 2.17 [0.69, 3.65] | | | + | + | + | + | + |
| Bishop 2001 | 714 | 277 | 12 | 491 | 113 | 12 | 23.9% | 1.02 [0.16, 1.88] | + | + | + | + | + | + | + |
| Langfort 1996 | 241.19 | 102.785 | 8 | 183.99 | 174.4008 | 8 | 19.3% | 0.38 [-0.61, 1.37] | | + | + | + | + | + | + |
| **Total (95% CI)** | | | **49** | | | **48** | **100.0%** | **0.81 [0.31, 1.31]** | | | | | | | |

Heterogeneity: Tau² = 0.08; Chi² = 5.27, df = 4 (P = 0.26); I² = 24%
Test for overall effect: Z = 3.18 (P = 0.001)

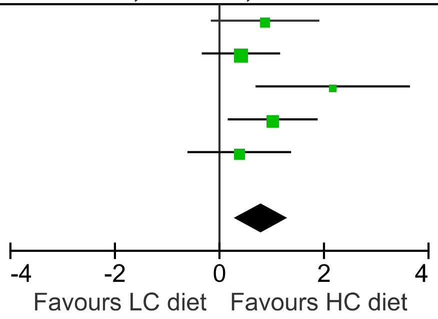


**F. Cortisol, 2 hr post-exercise (all studies: short-term, MP-LC diets & long-duration exercise only).**

| Study or Subgroup | LC diet Mean | LC diet SD | LC diet Total | HC diet Mean | HC diet SD | HC diet Total | Weight | Std. Mean Difference IV, Random, 95% CI | Risk of Bias A | B | C | D | E | F | G |
|---|---|---|---|---|---|---|---|---|---|---|---|---|---|---|---|
| Terink 2021 | 449.5 | 231.2344 | 14 | 301.7 | 237.2211 | 14 | 40.8% | 0.61 [-0.15, 1.37] | + | + | + | + | + | + | + |
| Bishop 2001 | 568 | 308 | 12 | 341 | 98 | 12 | 32.4% | 0.96 [0.11, 1.81] | + | + | + | + | + | + | + |
| Mitchell 1998 | 442.1 | 94.23587 | 10 | 348.9 | 92.33851 | 10 | 26.9% | 0.96 [0.02, 1.89] | + | + | + | + | + | + | + |
| **Total (95% CI)** | | | **36** | | | **36** | **100.0%** | **0.82 [0.33, 1.30]** | | | | | | | |

Heterogeneity: Tau² = 0.00; Chi² = 0.47, df = 2 (P = 0.79); I² = 0%
Test for overall effect: Z = 3.30 (P = 0.0010)

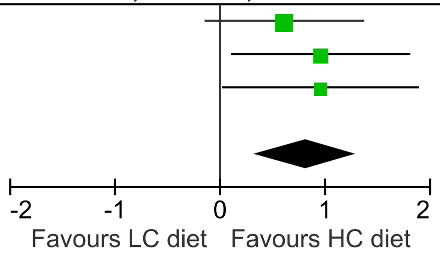

## G. Total testosterone, resting, long- vs short-term LC diets.

| | LC diet | | | HC diet | | | | Std. Mean Difference | Risk of Bias | | | | | | |
|---|---|---|---|---|---|---|---|---|---|---|---|---|---|---|---|
| Study or Subgroup | Mean | SD | Total | Mean | SD | Total | Weight | IV, Random, 95% CI | A | B | C | D | E | F | G |
| **2.1.1 Long-term LC diets (≥3 weeks)** | | | | | | | | | | | | | | | |
| Paoli 2021 | -2.44 | 4.09 | 9 | 0.31 | 4.91 | 10 | 8.4% | -0.58 [-1.50, 0.35] | + | | + | + | + | + | + |
| Toma 2009 | -3.72 | 3.52 | 8 | 0.51 | 3.31 | 9 | 7.3% | -1.18 [-2.23, -0.12] | + | | + | + | + | + | + |
| Volek 2002 | -0.4 | 10.1 | 12 | -0.2 | 6.9 | 8 | 8.6% | -0.02 [-0.92, 0.87] | | | + | + | + | + | − |
| Durkalec-Michalski 2021 | 5.84 | 0.88 | 11 | 5.59 | 1.09 | 11 | 9.1% | 0.24 [-0.60, 1.08] | | + | + | + | + | + | + |
| Michalczyk 2019 | 642.37 | 194.47 | 15 | 546.67 | 167.16 | 15 | 10.2% | 0.51 [-0.22, 1.24] | | + | + | + | + | + | ? |
| Zajac 2014 | 5.86 | 0.3 | 8 | 6.12 | 0.4 | 8 | 7.6% | -0.70 [-1.71, 0.32] | + | + | + | + | + | + | + |
| Krings 2021 | 5.48 | 2.08 | 9 | 5.93 | 1.89 | 9 | 8.4% | -0.22 [-1.14, 0.71] | | + | + | + | + | + | ? |
| Burke 2020 | -6.73286 | 3.580204 | 7 | 0.311111 | 3.255133 | 8 | 5.7% | -1.94 [-3.24, -0.65] | | ? | + | + | + | + | + |
| **Subtotal (95% CI)** | | | **79** | | | **78** | **65.4%** | **-0.39 [-0.90, 0.13]** | | | | | | | |
| Heterogeneity: Tau² = 0.31; Chi² = 16.52, df = 7 (P = 0.02); I² = 58% | | | | | | | | | | | | | | | |
| Test for overall effect: Z = 1.48 (P = 0.14) | | | | | | | | | | | | | | | |
| **2.1.2 Short-term LC diets (<3 weeks)** | | | | | | | | | | | | | | | |
| Anderson 1987 | 371 | 60.85228 | 7 | 468 | 89.95554 | 7 | 6.5% | -1.18 [-2.35, -0.01] | | + | + | + | + | + | + |
| Jaffe 2018 | -2.35 | 3.45 | 10 | 1.82 | 3.01 | 10 | 8.0% | -1.23 [-2.21, -0.26] | + | + | + | + | + | + | + |
| Burke 2021 | -2.6 | 4.8 | 7 | -1.7 | 3.5 | 6 | 7.0% | -0.20 [-1.29, 0.90] | | | + | + | + | + | + |
| Tsai 1993 | 25.7 | 5.8 | 4 | 23.4 | 5.2 | 4 | 5.2% | 0.36 [-1.05, 1.77] | | + | + | + | + | + | + |
| Langfort 2001 | 18.73 | 3.27 | 9 | 22.17 | 4.35 | 9 | 7.9% | -0.85 [-1.83, 0.12] | | + | + | + | + | + | + |
| **Subtotal (95% CI)** | | | **37** | | | **36** | **34.6%** | **-0.71 [-1.26, -0.17]** | | | | | | | |
| Heterogeneity: Tau² = 0.07; Chi² = 4.89, df = 4 (P = 0.30); I² = 18% | | | | | | | | | | | | | | | |
| Test for overall effect: Z = 2.56 (P = 0.01) | | | | | | | | | | | | | | | |
| **Total (95% CI)** | | | **116** | | | **114** | **100.0%** | **-0.48 [-0.87, -0.09]** | | | | | | | |
| Heterogeneity: Tau² = 0.25; Chi² = 23.72, df = 12 (P = 0.02); I² = 49% | | | | | | | | | | | | | | | |
| Test for overall effect: Z = 2.43 (P = 0.01) | | | | | | | | | | | | | | | |
| Test for subgroup differences: Chi² = 0.74, df = 1 (P = 0.39), I² = 0% | | | | | | | | | | | | | | | |

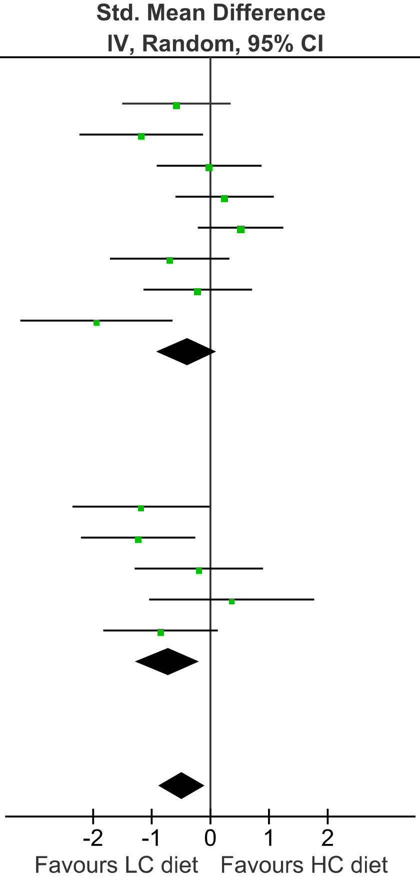

## H. Total testosterone, resting, MP- vs HP-LC diets.

| | LC diet | | | HC diet | | | | Std. Mean Difference | Risk of Bias | | | | | | |
|---|---|---|---|---|---|---|---|---|---|---|---|---|---|---|---|
| **Study or Subgroup** | **Mean** | **SD** | **Total** | **Mean** | **SD** | **Total** | **Weight** | **IV, Random, 95% CI** | **A** | **B** | **C** | **D** | **E** | **F** | **G** |
| **2.2.1 MP-LC diets (<35%)** | | | | | | | | | | | | | | | |
| Paoli 2021 | -2.44 | 4.09 | 9 | 0.31 | 4.91 | 10 | 8.4% | -0.58 [-1.50, 0.35] | + | | + | + | + | + | + |
| Toma 2009 | -3.72 | 3.52 | 8 | 0.51 | 3.31 | 9 | 7.3% | -1.18 [-2.23, -0.12] | + | | + | + | + | + | + |
| Volek 2002 | -0.4 | 10.1 | 12 | -0.2 | 6.9 | 8 | 8.6% | -0.02 [-0.92, 0.87] | | | + | + | + | + | − |
| Durkalec-Michalski 2021 | 5.84 | 0.88 | 11 | 5.59 | 1.09 | 11 | 9.1% | 0.24 [-0.60, 1.08] | | + | + | + | + | + | + |
| Michalczyk 2019 | 642.37 | 194.47 | 15 | 546.67 | 167.16 | 15 | 10.2% | 0.51 [-0.22, 1.24] | | + | + | + | + | + | ? |
| Zajac 2014 | 5.86 | 0.3 | 8 | 6.12 | 0.4 | 8 | 7.6% | -0.70 [-1.71, 0.32] | + | + | + | + | + | + | + |
| Krings 2021 | 5.48 | 2.08 | 9 | 5.93 | 1.89 | 9 | 8.4% | -0.22 [-1.14, 0.71] | | + | + | + | + | + | ? |
| Burke 2020 | -6.73286 | 3.580204 | 7 | 0.311111 | 3.255133 | 8 | 5.7% | -1.94 [-3.24, -0.65] | | ? | + | + | + | + | + |
| Burke 2021 | -2.6 | 4.8 | 7 | -1.7 | 3.5 | 6 | 7.0% | -0.20 [-1.29, 0.90] | | | + | + | + | + | + |
| Tsai 1993 | 25.7 | 5.8 | 4 | 23.4 | 5.2 | 4 | 5.2% | 0.36 [-1.05, 1.77] | | + | + | + | + | + | + |
| **Subtotal (95% CI)** | | | **90** | | | **88** | **77.6%** | **-0.31 [-0.74, 0.13]** | | | | | | | |
| Heterogeneity: Tau² = 0.23; Chi² = 17.26, df = 9 (P = 0.04); I² = 48% | | | | | | | | | | | | | | | |
| Test for overall effect: Z = 1.39 (P = 0.17) | | | | | | | | | | | | | | | |
| **2.2.2 HP-LC diets (≥35%)** | | | | | | | | | | | | | | | |
| Anderson 1987 | 371 | 60.85228 | 7 | 468 | 89.95554 | 7 | 6.5% | -1.18 [-2.35, -0.01] | | + | + | + | + | + | + |
| Jaffe 2018 | -2.35 | 3.45 | 10 | 1.82 | 3.01 | 10 | 8.0% | -1.23 [-2.21, -0.26] | + | + | + | + | + | + | + |
| Langfort 2001 | 18.73 | 3.27 | 9 | 22.17 | 4.35 | 9 | 7.9% | -0.85 [-1.83, 0.12] | | + | + | + | + | + | + |
| **Subtotal (95% CI)** | | | **26** | | | **26** | **22.4%** | **-1.08 [-1.67, -0.48]** | | | | | | | |
| Heterogeneity: Tau² = 0.00; Chi² = 0.34, df = 2 (P = 0.85); I² = 0% | | | | | | | | | | | | | | | |
| Test for overall effect: Z = 3.56 (P = 0.0004) | | | | | | | | | | | | | | | |
| **Total (95% CI)** | | | **116** | | | **114** | **100.0%** | **-0.48 [-0.87, -0.09]** | | | | | | | |
| Heterogeneity: Tau² = 0.25; Chi² = 23.72, df = 12 (P = 0.02); I² = 49% | | | | | | | | | | | | | | | |
| Test for overall effect: Z = 2.43 (P = 0.01) | | | | | | | | | | | | | | | |
| Test for subgroup differences: Chi² = 4.25, df = 1 (P = 0.04), I² = 76.5% | | | | | | | | | | | | | | | |

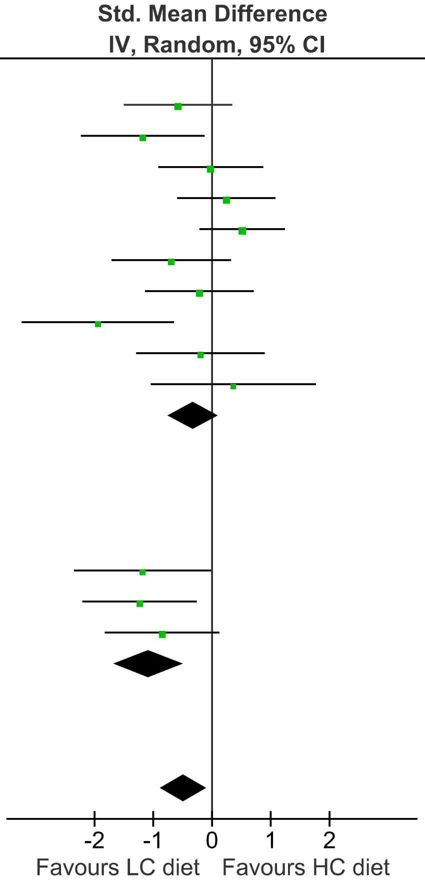

## I. Total testosterone, 0 hr post-exercise, long- vs short-term LC diets.

| Study or Subgroup | LC diet Mean | LC diet SD | LC diet Total | HC diet Mean | HC diet SD | HC diet Total | Weight | Std. Mean Difference IV, Random, 95% CI | A | B | C | D | E | F | G |
|---|---|---|---|---|---|---|---|---|---|---|---|---|---|---|---|
| **4.1.1 Long-term LC diets (≥3 weeks)** | | | | | | | | | | | | | | | |
| Durkalec-Michalski 2021 | 6.25 | 0.67 | 11 | 5.9 | 0.75 | 11 | 35.7% | 0.47 [-0.38, 1.32] | | + | + | + | + | + | + |
| Zajac 2014 | 8.14 | 0.6 | 8 | 7.91 | 0.5 | 8 | 32.2% | 0.39 [-0.60, 1.39] | + | + | + | + | + | + | + |
| **Subtotal (95% CI)** | | | **19** | | | **19** | **67.9%** | **0.44 [-0.21, 1.09]** | | | | | | | |
| Heterogeneity: Tau² = 0.00; Chi² = 0.01, df = 1 (P = 0.90); I² = 0% | | | | | | | | | | | | | | | |
| Test for overall effect: Z = 1.34 (P = 0.18) | | | | | | | | | | | | | | | |
| **4.1.2 Short-term LC diets (<3 weeks)** | | | | | | | | | | | | | | | |
| Langfort 2001 | 24.39 | 4.92 | 9 | 29.25 | 4.26 | 9 | 32.1% | -1.01 [-2.00, -0.01] | | + | + | + | + | + | + |
| **Subtotal (95% CI)** | | | **9** | | | **9** | **32.1%** | **-1.01 [-2.00, -0.01]** | | | | | | | |
| Heterogeneity: Not applicable | | | | | | | | | | | | | | | |
| Test for overall effect: Z = 1.98 (P = 0.05) | | | | | | | | | | | | | | | |
| **Total (95% CI)** | | | **28** | | | **28** | **100.0%** | **-0.03 [-0.95, 0.89]** | | | | | | | |
| Heterogeneity: Tau² = 0.43; Chi² = 5.71, df = 2 (P = 0.06); I² = 65% | | | | | | | | | | | | | | | |
| Test for overall effect: Z = 0.06 (P = 0.95) | | | | | | | | | | | | | | | |
| Test for subgroup differences: Chi² = 5.70, df = 1 (P = 0.02), I² = 82.5% | | | | | | | | | | | | | | | |

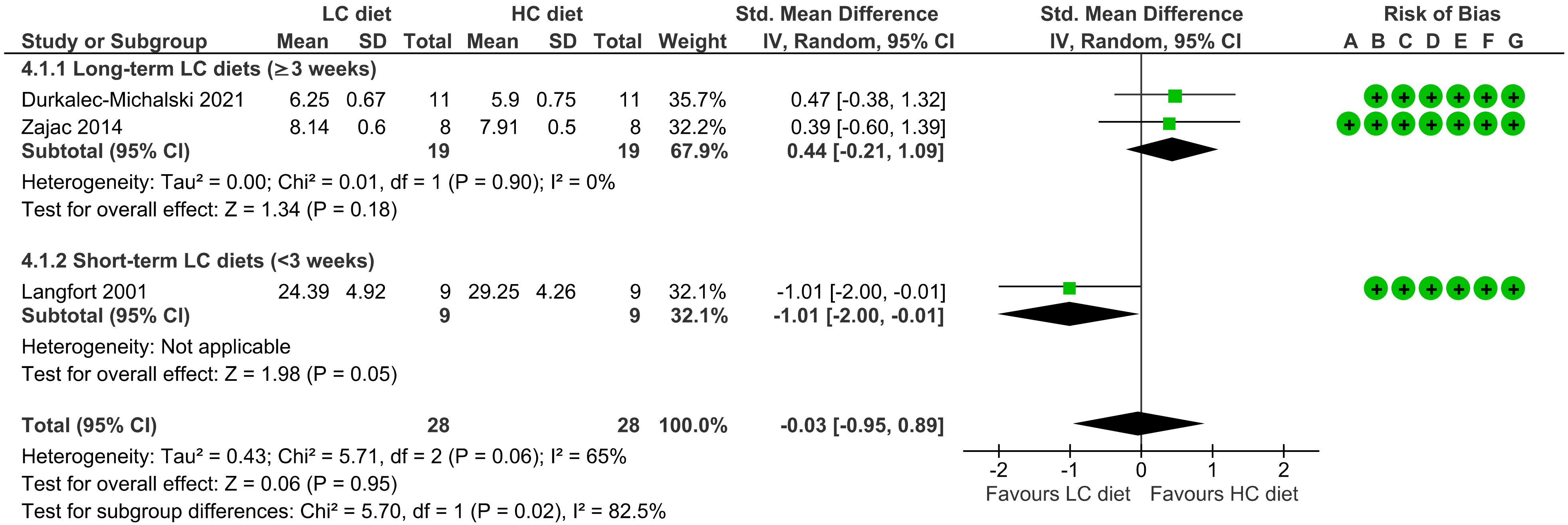


**Figure 2.** Forest plots with subgroup analyses, showing standardized mean differences of LC vs HC diets.
HC: high-carbohydrate; HP: high-protein; LC: low-carbohydrate; MP: moderate-protein.

<u>Risk of bias legend</u>

(A) Bias due to randomization process
(B) Bias due to period and carryover effects
(C) Bias due to deviations from intended interventions
(D) Bias due to missing outcome data
(E) Bias due to measurement of outcome
(F) Bias due to selection of reported result
(G) Bias due to confounding variables

**A. Cortisol, resting, long- vs short-term LC diets.**

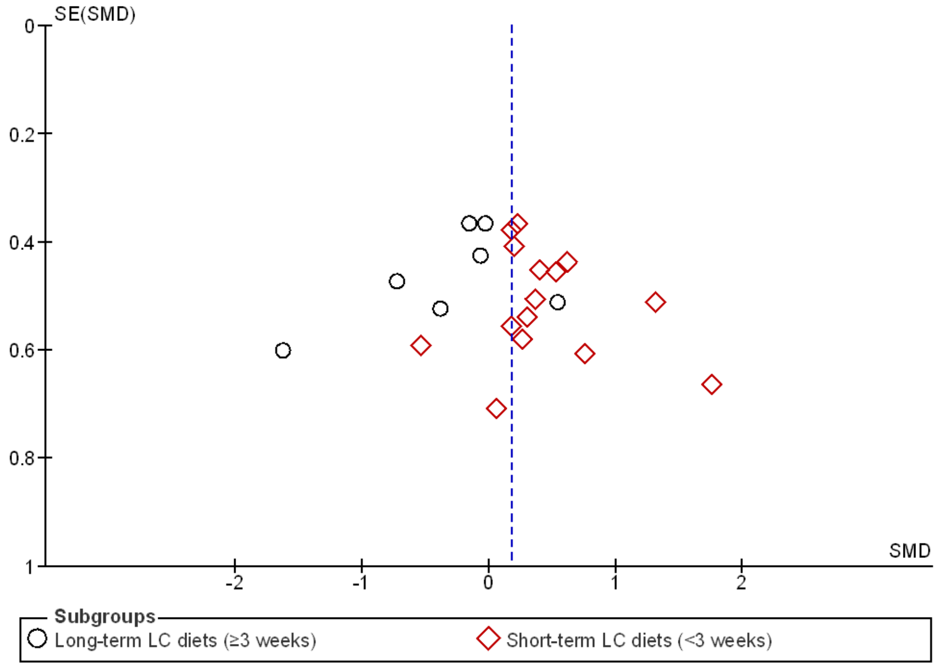


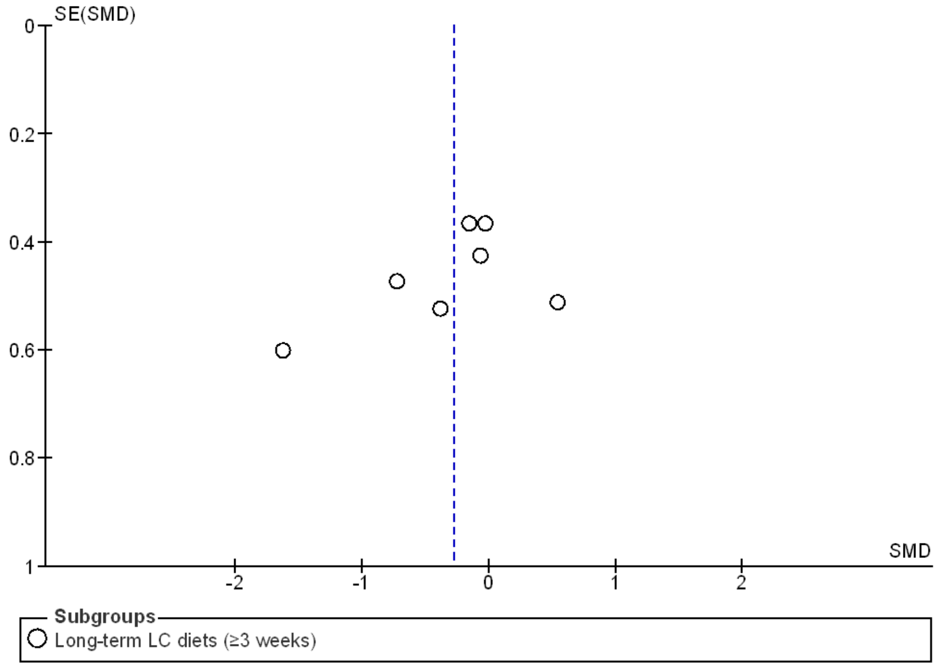


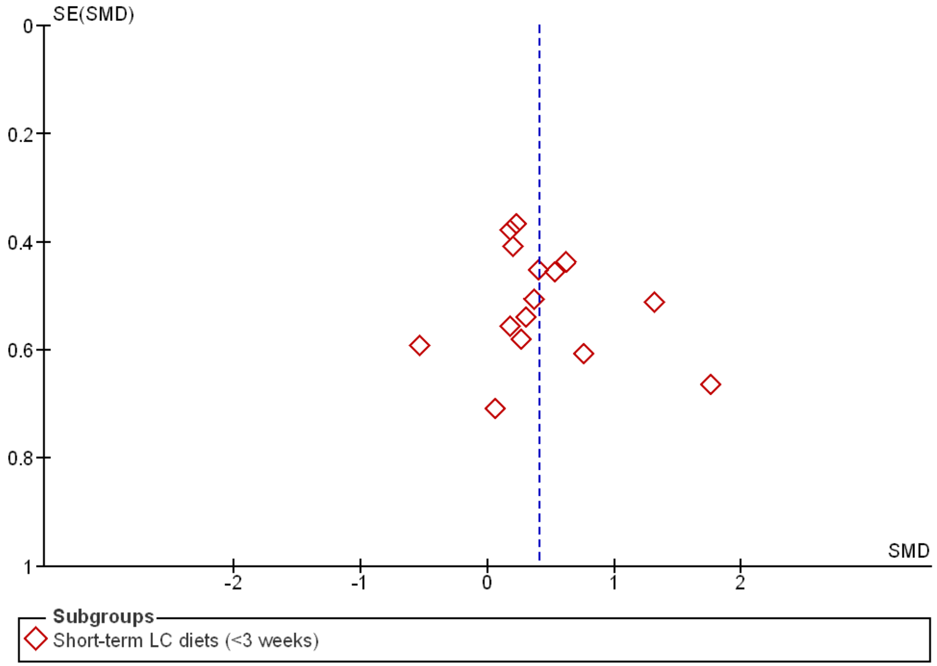

**B. Cortisol, 0 hr post-exercise, long- vs short-duration exercise.**

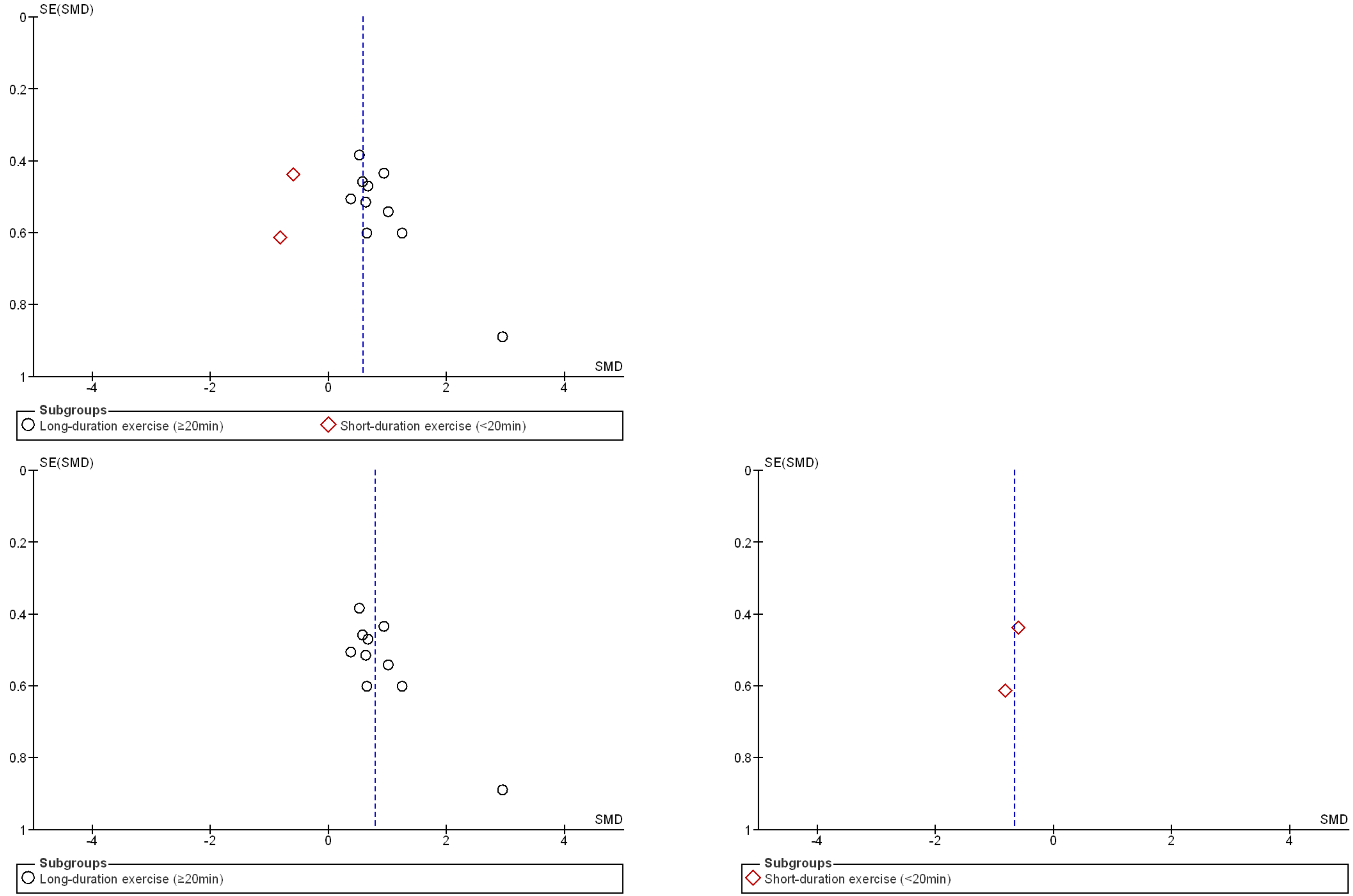

**C. Cortisol, 1 hr post-exercise.**

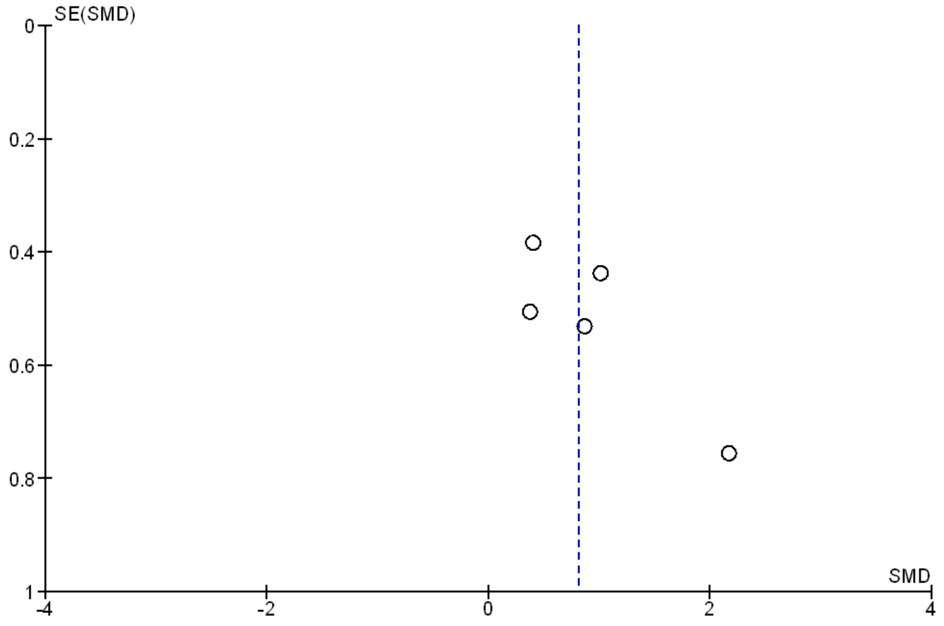


**D. Cortisol, 2 hr post-exercise.**

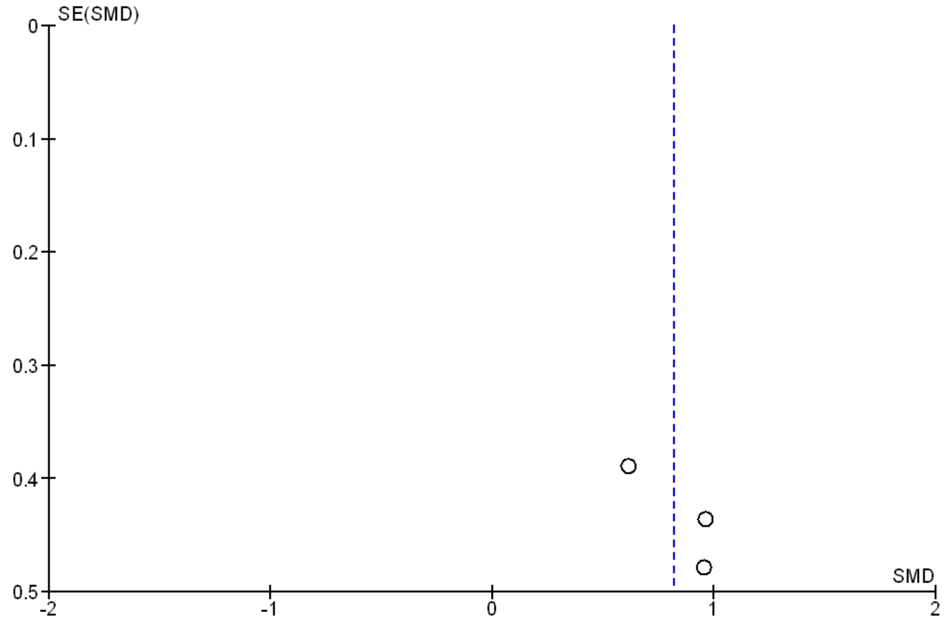

**E. Total testosterone, resting, MP- vs HP-LC diets.**

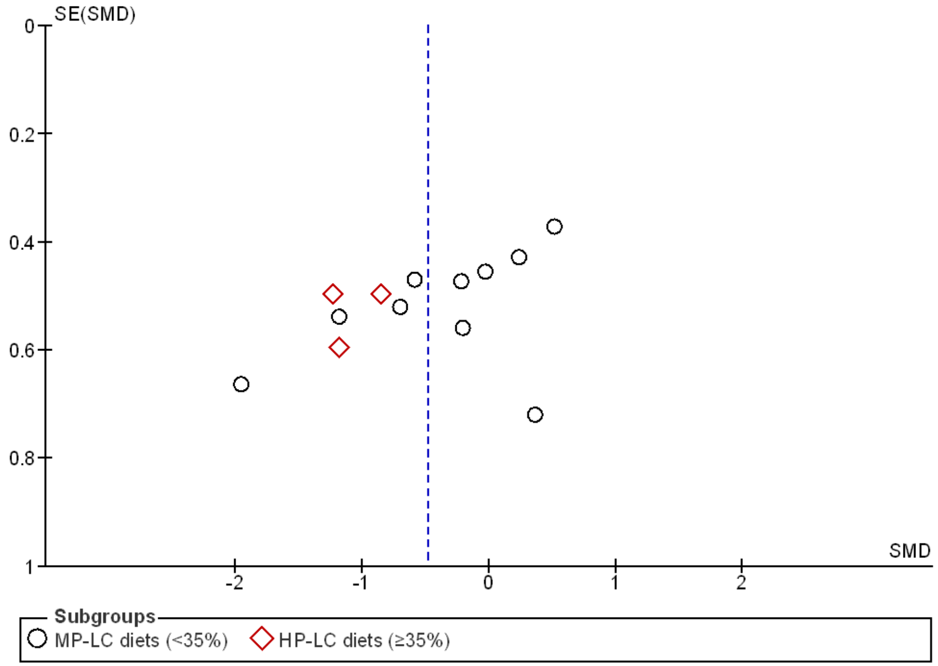


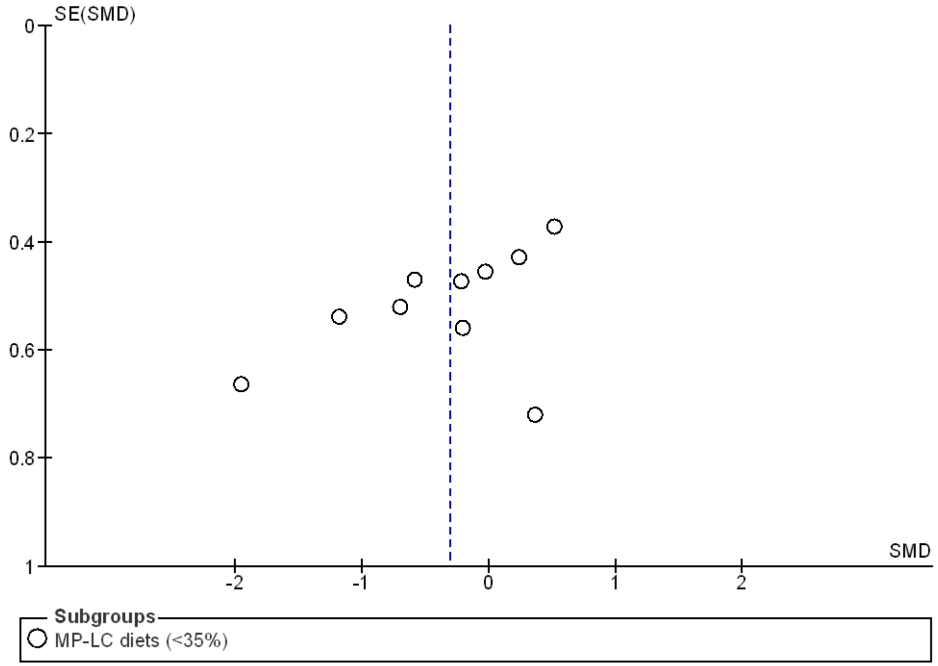


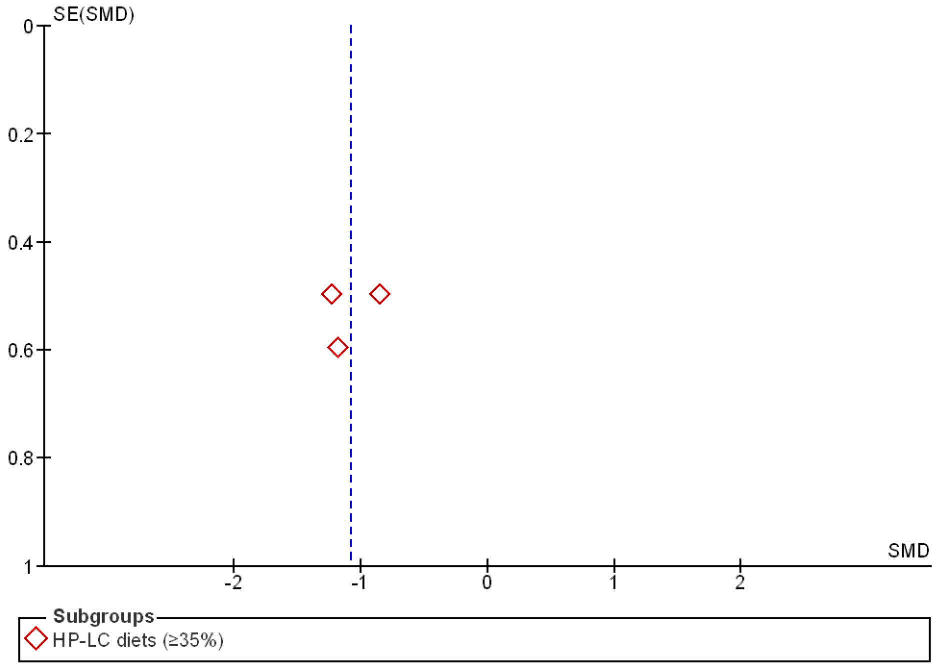

**F. Total testosterone, 0 hr post-exercise, long- vs short-term LC diets.**

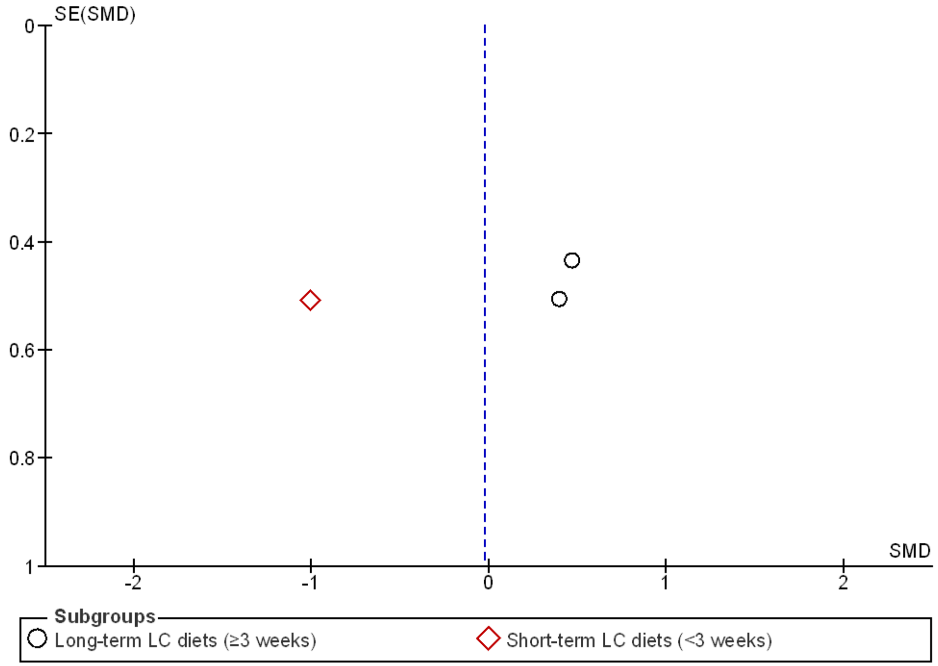


**Figure 3.** Funnel plots of outcomes, with subgroups that explain heterogeneity. HC: high-carbohydrate; HP: high-protein; LC: low-carbohydrate; MP: moderate-protein; SE: standard error; SMD: standardized mean difference.

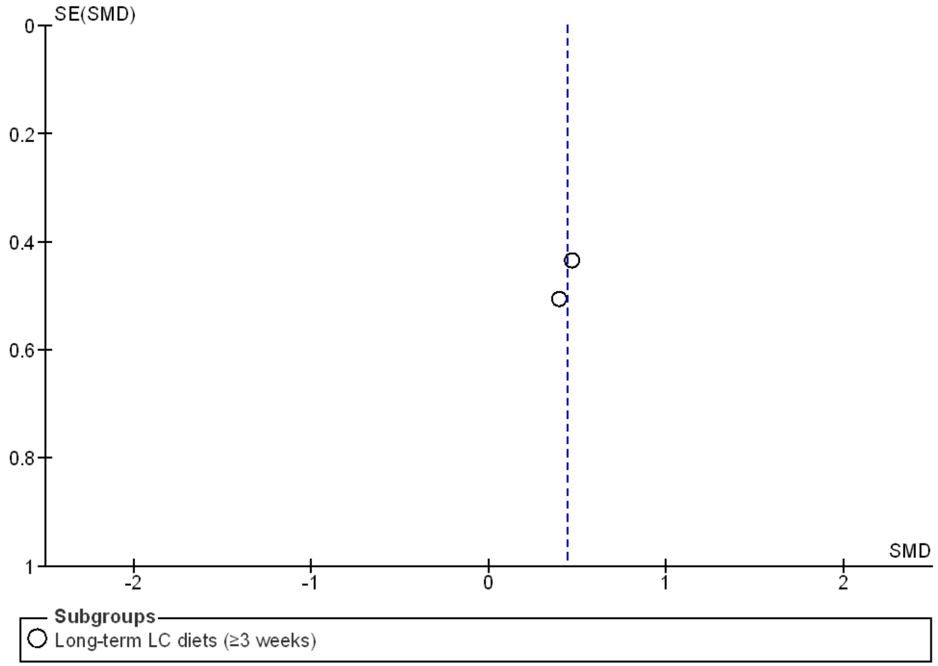


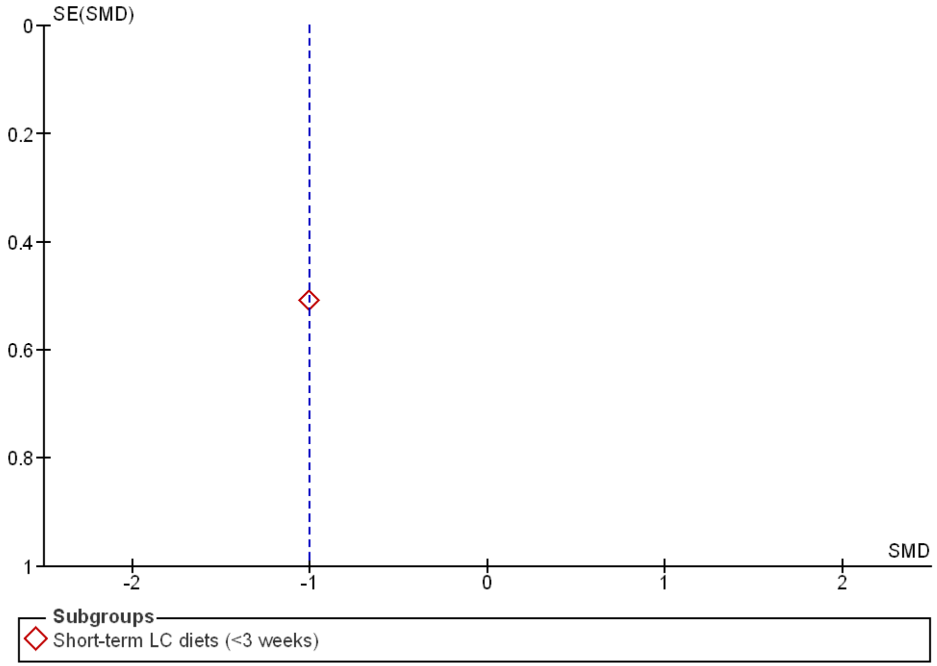

**Table 9.** Sensitivity analyses.

| Sensitivity analysis, outcome & subgroup[a] | Participants (studies) | SMD [95% CI] | P-value (Z test) | $I^2$ (%) | P-value ($Chi^2$ test) | Significantly different to original result (yes/no)[b] |
|---|---|---|---|---|---|---|
| <u>High and medium bias studies excluded</u> | | | | | | |
| Cortisol, resting | | | | | | |
| All studies | 170 (17) | 0.34 [0.07, 0.62] | 0.01 | 26 | 0.15 | no |
| Long-term LC diets | 27 (3) | -0.33 [-1.48, 0.81] | 0.57 | 75 | 0.02 | no |
| Short-term LC diets | 143 (14) | 0.45 [0.2, 0.71] | <0.01 | 0 | 0.76 | no |
| Test for subgroup differences | | | | 42.3 | 0.19 | no |
| Cortisol, 0 hr post-exercise | | | | | | |
| All studies | 117 (11) | 0.58 [0.13, 1.03] | 0.01 | 55 | 0.01 | no |
| Long-duration exercise | 100 (9) | 0.79 [0.46, 1.12] | <0.01 | 3 | 0.41 | no |
| Short-duration exercise | NA[c] | NA | NA | NA | NA | NA |
| Test for subgroup differences | | | | 92.8 | <0.01 | no |
| Cortisol, 1 hr post-exercise | | | | | | |
| All studies | NA | NA | NA | NA | NA | NA |
| Cortisol, 2 hr post-exercise | | | | | | |
| All studies | NA | NA | NA | NA | NA | NA |
| Total testosterone, resting | | | | | | |
| All studies | 98 (9) | -0.6 [-0.99, -0.21] | <0.01 | 22 | 0.25 | no |
| MP-LC diets | 72 (6) | -0.36 [-0.82, 0.1] | 0.13 | 17 | 0.3 | no |
| HP-LC diets | NA | NA | NA | NA | NA | NA |
| Test for subgroup differences | | | | 71.8 | 0.06 | no |

| Sensitivity analysis, outcome & subgroup[a] | Participants (studies) | SMD [95% CI] | P-value (Z test) | $I^2$ (%) | P-value ($Chi^2$ test) | Significantly different to original result (yes/no)[b] |
|---|---|---|---|---|---|---|
| Total testosterone, 0 hr post-exercise | | | | | | |
| All studies | NA | NA | NA | NA | NA | NA |
| Long-term LC diets | NA | NA | NA | NA | NA | NA |
| Short-term LC diets | NA | NA | NA | NA | NA | NA |
| Test for subgroup differences | | | | NA | NA | NA |
| | | | | | | |
| <u>Non-randomized studies excluded</u> | | | | | | |
| Cortisol, resting | | | | | | |
| All studies | 96 (9) | 0.22 [-0.27, 0.7] | 0.38 | 54 | 0.03 | no |
| Long-term LC diets | 16 (2) | -0.52 [-2.64, 1.61] | 0.64 | 87 | <0.01 | no |
| Short-term LC diets | 80 (7) | 0.38 [0, 0.76] | 0.05 | 10 | 0.35 | no |
| Test for subgroup differences | | | | 0 | 0.42 | no |
| Cortisol, 0 hr post-exercise | | | | | | |
| All studies | 90 (8) | 0.56 [0.22, 0.89] | <0.01 | 0 | 0.46 | yes (reduced heterogeneity) |
| Long-duration exercise | 84 (7) | 0.67 [0.32, 1.02] | <0.01 | 0 | 0.97 | no |
| Short-duration exercise | 6 (1) | -0.81 [-2.01, 0.39] | 0.18 | NA[d] | NA | no |
| Test for subgroup differences | | | | 81.5 | 0.02 | no |
| Cortisol, 1 hr post-exercise | | | | | | |
| All studies | 34 (3) | 0.72 [0.22, 1.22] | <0.01 | 0 | 0.55 | no |
| Cortisol, 2 hr post-exercise | | | | | | |
| All studies | NA | NA | NA | NA | NA | NA |

| Sensitivity analysis, outcome & subgroup[a] | Participants (studies) | SMD [95% CI] | P-value (Z test) | $I^2$ (%) | P-value ($Chi^2$ test) | Significantly different to original result (yes/no)[b] |
|---|---|---|---|---|---|---|
| Total testosterone, resting | | | | | | |
| All studies | 54 (4) | -0.91 [-1.4, -0.41] | <0.01 | 0 | 0.72 | yes (stronger effect and reduced heterogeneity) |
| MP-LC diets | 44 (3) | -0.79 [-1.37, -0.22] | <0.01 | 0 | 0.69 | yes (stronger effect, p <0.05, and reduced heterogeneity) |
| HP-LC diets | 10 (1) | -1.23 [-2.21, -0.26] | 0.01 | NA | NA | no |
| Test for subgroup differences | | | | 0 | 0.45 | yes (no subgroup effect) |
| Total testosterone, 0 hr post-exercise | | | | | | |
| All studies | 8 (1) | 0.39 [-0.6, 1.39] | 0.44 | NA | NA | no |
| Long-term LC diets | 8 (1) | 0.39 [-0.6, 1.39] | 0.44 | NA | NA | no |
| Short-term LC diets | NA | NA | NA | NA | NA | NA |
| Test for subgroup differences | | | | NA | NA | NA |
| <u>Studies using carbohydrate supplements during exercise on HC diets excluded</u> | | | | | | |
| Cortisol, 0 hr post-exercise | | | | | | |
| All studies | 98 (9) | 0.41 [-0.01, 0.83] | 0.06 | 40 | 0.1 | yes (weaker effect) |
| Long-duration exercise | 81 (7) | 0.7 [0.34, 1.06] | <0.01 | 0 | 0.94 | no |
| Short-duration exercise | NA | NA | NA | NA | NA | NA |
| Test for subgroup differences | | | | 91.4 | <0.01 | no |
| Cortisol, 1 hr post-exercise | | | | | | |
| All studies | 34 (3) | 0.6 [0.11, 1.09] | 0.02 | 0 | 0.51 | yes (weaker effect) |
| Cortisol, 2 hr post-exercise | | | | | | |
| All studies | 26 (2) | 0.77 [0.2, 1.33] | <0.01 | 0 | 0.55 | no |

| Sensitivity analysis, outcome & subgroup[a] | Participants (studies) | SMD [95% CI] | P-value (Z test) | $I^2$ (%) | P-value ($Chi^2$ test) | Significantly different to original result (yes/no)[b] |
|---|---|---|---|---|---|---|
| Total testosterone, 0 hr post-exercise | | | | | | |
| All studies | NA | NA | NA | NA | NA | NA |
| Long-term LC diets | NA | NA | NA | NA | NA | NA |
| Short-term LC diets | NA | NA | NA | NA | NA | NA |
| Test for subgroup differences | | | | NA | NA | NA |
| <u>Using alternative HC diets from studies with two eligible HC diets</u> | | | | | | |
| Cortisol, resting | | | | | | |
| All studies | 245 (22) | 0.17 [-0.08, 0.42] | 0.18 | 33 | 0.07 | no |
| Long-term LC diets | NA | NA | NA | NA | NA | NA |
| Short-term LC diets | 155 (15) | 0.4 [0.15, 0.65] | <0.01 | 0 | 0.56 | no |
| Test for subgroup differences | | | | 86.3 | <0.01 | no |
| Cortisol, 0 hr post-exercise | | | | | | |
| All studies | NA | NA | NA | NA | NA | NA |
| Long-duration exercise | NA | NA | NA | NA | NA | NA |
| Short-duration exercise | NA | NA | NA | NA | NA | NA |
| Test for subgroup differences | | | | NA | NA | NA |
| Cortisol, 1 hr post-exercise | | | | | | |
| All studies | NA | NA | NA | NA | NA | NA |
| Cortisol, 2 hr post-exercise | | | | | | |
| All studies | NA | NA | NA | NA | NA | NA |

| Sensitivity analysis, outcome & subgroup[a] | Participants (studies) | SMD [95% CI] | P-value (Z test) | $I^2$ (%) | P-value ($Chi^2$ test) | Significantly different to original result (yes/no)[b] |
|---|---|---|---|---|---|---|
| Total testosterone, resting | | | | | | |
| All studies | 155 (13) | -0.48 [-0.87, -0.08] | 0.02 | 50 | 0.02 | no |
| MP-LC diets | 129 (10) | -0.3 [-0.74, 0.14] | 0.18 | 49 | 0.04 | no |
| HP-LC diets | NA | NA | NA | NA | NA | NA |
| Test for subgroup differences | | | | 76.7 | 0.04 | no |
| Total testosterone, 0 hr post-exercise | | | | | | |
| All studies | NA | NA | NA | NA | NA | NA |
| Long-term LC diets | NA | NA | NA | NA | NA | NA |
| Short-term LC diets | NA | NA | NA | NA | NA | NA |
| Test for subgroup differences | | | | NA | NA | NA |
| <u>One study at a time excluded & analysis rerun</u> (ranges presented) | | | | | | |
| Cortisol, resting | | | | | | |
| All studies | 225 - 241 (21) | 0.13 [-0.09, 0.36] - 0.23 [0.02, 0.44] | 0.03 - 0.28 | 7 - 35 | 0.06 - 0.37 | no |
| Long-term LC diets | 70 - 82 (6) | -0.13 [-0.47, 0.22] - -0.37 [-0.79, 0.04] | 0.07 – 0.47 | 0 - 46 | 0.1 – 0.6 | no |
| Short-term LC diets | 135 - 151 (14) | 0.35 [0.09, 0.61] - 0.45 [0.2, 0.71] | <0.01 - <0.01 | 0 - 0 | 0.55 – 0.88 | no |
| Test for subgroup differences | | | | 81 – 90.2 | <0.01 - 0.02 | no |

| Sensitivity analysis, outcome & subgroup[a] | Participants (studies) | SMD [95% CI] | P-value (Z test) | $I^2$ (%) | P-value ($Chi^2$ test) | Significantly different to original result (yes/no)[b] |
|---|---|---|---|---|---|---|
| Cortisol, 0 hr post-exercise | | | | | | |
| All studies | 109 – 123 (11) | 0.47 [0.12, 0.83] - 0.7 [0.32, 1.07] | <0.01 – 0.02 | 32 - 55 | 0.01 - 0.15 | no |
| Long-duration exercise | 92 - 105 (9) | 0.71 [0.39, 1.03] - 0.84 [0.49, 1.18] | <0.01 - <0.01 | 0 - 3 | 0.41 – 0.98 | no |
| Short-duration exercise | 6 – 11 (1) | -0.6 [-1.46, 0.26] - -0.81 [-2.01, 0.39] | 0.17 - 0.18 | NA | NA | no |
| Test for subgroup differences | | | | 84.2 – 93.1 | <0.01 – 0.01 | no |
| Cortisol, 1 hr post-exercise | | | | | | |
| All studies | 41 - 47 (4) | 0.65 [0.21, 1.09] - 0.97 [0.37, 1.57] | <0.01 - 0.02 | 0 - 43 | 0.16 – 0.67 | no |
| Cortisol, 2 hr post-exercise | | | | | | |
| All studies | 22 – 26 (2) | 0.75 [0.16, 1.34] - 0.96 [0.33, 1.59] | <0.01 – 0.01 | 0 - 0 | 0.55 - 1 | no |
| Total testosterone, resting | | | | | | |
| All studies | 135 - 151 (12) | -0.59 [-0.95, -0.22] - -0.38 [-0.75, -0.02] | <0.01 - 0.04 | 33 - 53 | 0.01 – 0.12 | no |
| MP-LC diets | 109 – 125 (9) | -0.16 [-0.52, 0.2] - -0.42 [-0.85, 0] | 0.05 - 0.4 | 22 - 54 | 0.03 – 0.25 | no |
| HP-LC diets | 16 – 19 (2) | -0.99 [-1.74, -0.24] - -1.21 [-1.96, -0.46] | <0.01 – 0.01 | 0 - 0 | 0.59 – 0.95 | no |
| Test for subgroup differences | | | | 58.1 – 85.3 | <0.01 - 0.12 | no |

| Sensitivity analysis, outcome & subgroup[a] | Participants (studies) | SMD [95% CI] | P-value (Z test) | $I^2$ (%) | P-value ($Chi^2$ test) | Significantly different to original result (yes/no)[b] |
|---|---|---|---|---|---|---|
| Total testosterone, 0 hr post-exercise | | | | | | |
| All studies | 17 – 20 (2) | -0.31 [-1.68, 1.07] - 0.44 [-0.21, 1.09] | 0.18 - 0.74 | 0 - 80 | 0.03 – 0.9 | no |
| Long-term LC diets | 8 – 11 (1) | 0.39 [-0.6, 1.39] - 0.47 [-0.38, 1.32] | 0.27 - 0.44 | NA | NA | no |
| Short-term LC diets | NA | NA | NA | NA | NA | NA |
| Test for subgroup differences | | | | 73.7 - 79.6 | 0.03 – 0.05 | no |

[a] Long-term (≥3 weeks), short-term (<3 weeks), long-duration exercise (≥20 min), short-duration exercise (<20 min), MP (<35% protein), HP (≥35% protein).

[b] This was a qualitative not quantitative judgement.

[c] NA as the sensitivity analyses were identical to original analyses (i.e. the outcome or subgroup contains no high/ medium bias, non-randomized, carbohydrate supplement, or alternative HC diet studies).

[d] NA for $I^2$ and $Chi^2$ tests in subgroups containing only one study.

CI: confidence interval; HC: high-carbohydrate; HP: high-protein; LC: low-carbohydrate; MP: moderate-protein; NA: not applicable; SMD: standardized mean difference.

**References**

Alvares AP, Anderson KE, Conney AH, et al. (1976) Interactions between nutritional factors and drug biotransformations in man. *Proceedings of the National Academy of Sciences of the United States of America* 73(7): 2501–2504.

Anderson KE, Rosner W, Khan MS, et al. (1987) Diet-hormone interactions: protein/carbohydrate ratio alters reciprocally the plasma levels of testosterone and cortisol and their respective binding globulins in man. *Life Sciences* 40(18): 1761–1768.

Anderson RA, Bryden NA, Polansky MM, et al. (1991) Effects of carbohydrate loading and underwater exercise on circulating cortisol, insulin and urinary losses of chromium and zinc. *European Journal of Applied Physiology and Occupational Physiology* 63(2): 146–150.

Beall J (2021) Beall's list of potential predatory journals and publishers. Available at: https://beallslist.net/ (accessed 5 August 2021).

Bishop NC, Walsh NP, Haines DL, et al. (2001a) Pre-exercise carbohydrate status and immune responses to prolonged cycling: I. effect on neutrophil degranulation. *International Journal of Sport Nutrition and Exercise Metabolism* 11(4): 490–502.

Bishop NC, Walsh NP, Haines DL, et al. (2001b) Pre-exercise carbohydrate status and immune responses to prolonged cycling: II. effect on plasma cytokine concentration. *International Journal of Sport Nutrition and Exercise Metabolism* 11(4): 503–512.

Bisschop PH, Pereira Arias AM, Ackermans MT, et al. (2000) The effects of carbohydrate variation in isocaloric diets on glycogenolysis and gluconeogenesis in healthy men. *The Journal of Clinical Endocrinology and Metabolism* 85(5): 1963–1967.

Burke L (2018) The effect of low energy availability and low carbohydrate availability on hormone status, metabolism and performance in elite race walkers ('Supernova 4"). Available at: https://anzctr.org.au/Trial/Registration/TrialReview.aspx?ACTRN=12618001974291 (accessed 2 August 2021).

Burke L (2019) Supernova 2: dietary periodisation and de-adaptation to support training outcomes in elite race walkers. Available at: https://anzctr.org.au/Trial/Registration/TrialReview.aspx?ACTRN=12619000794101 (accessed 31 July 2021).

Burke LM, Ross ML, Garvican-Lewis LA, et al. (2017) Low carbohydrate, high fat diet impairs exercise economy and negates the performance benefit from intensified training in elite race walkers. *The Journal of Physiology* 595(9): 2785–2807.

Burke LM, Sharma AP, Heikura IA, et al. (2020) Crisis of confidence averted: impairment of exercise economy and performance in elite race walkers by ketogenic low carbohydrate, high fat (LCHF) diet is reproducible. *PLOS One* 15(6): e0234027.

Burke LM, Whitfield J, Heikura IA, et al. (2021) Adaptation to a low carbohydrate high fat diet is rapid but impairs endurance exercise metabolism and performance despite enhanced glycogen availability. *The Journal of Physiology* 599(3): 771–790.

Cohen E, Cragg M, DeFonseka J, et al. (2015) Statistical review of US macronutrient consumption data, 1965-2011: Americans have been following dietary guidelines, coincident with the rise in obesity. *Nutrition* 31(5): 727–732.

Deeks JJ and Higgins JPT (2021) Chapter 10 - S1. Supplementary material: statistical algorithms in review manager 5.1. In: Higgins JPT, Thomas J, Chandler J, et al. (eds) *Cochrane Handbook for Systematic Reviews of Interventions*. 6.2. London: Cochrane. Availiable at: https://training.cochrane.org/handbook/current/chapter-10-statistical-algorithms-revman-5-1 (accessed 19 August 2021)

Duke Jr. JW (2008) *Influence of exercise training on the free testosterone to cortisol ratio*. Master's Thesis, University of North Carolina at Chapel Hill, USA.

Durkalec-Michalski K, Nowaczyk PM and Siedzik K (2019) Effect of a four-week ketogenic diet on exercise metabolism in CrossFit-trained athletes. *Journal of the International Society of Sports Nutrition* 16(1): 16.

Durkalec-Michalski K, Nowaczyk PM, Główka N, et al. (2021) Is a four-week ketogenic diet an effective nutritional strategy in CrossFit-trained female and male athletes? *Nutrients* 13(3): 864.

Ebbeling CB, Feldman HA, Klein GL, et al. (2018) Effects of a low carbohydrate diet on energy expenditure during weight loss maintenance: randomized trial. *BMJ (Clinical Research Ed.)* 363: k4583.

Ebbeling CB, Bielak L, Lakin PR, et al. (2020) Energy requirement is higher during weight-loss maintenance in adults consuming a low- compared with high-carbohydrate diet. *The Journal of Nutrition* 150(8): 2009–2015.

Galbo H, Holst JJ and Christensen NJ (1979) The effect of different diets and of insulin on the hormonal response to prolonged exercise. *Acta Physiologica Scandinavica* 107(1): 19–32.

Gleeson M, Blannin AK, Walsh NP, et al. (1998) Effect of low- and high-carbohydrate diets on the plasma glutamine and circulating leukocyte responses to exercise. *International Journal of Sport Nutrition* 8(1): 49–59.

Gray CG, Kolterman OG and Cutler DC (1990) The effect of a three-week adaptation to a low carbohydrate/high fat diet on metabolism and cognitive performance. Report, Naval Health Research Center, USA, April.

Heikura IA, Burke LM, Hawley JA, et al. (2019) A short-term ketogenic diet impairs markers of bone health in response to exercise. *Frontiers in Endocrinology* 10: 880.

Hélie R, Lavoie JM and Cousineau D (1985) Effects of a 24-h carbohydrate-poor diet on metabolic and hormonal responses during prolonged glucose-infused leg exercise. *European Journal of Applied Physiology and Occupational Physiology* 54(4): 420–426.

Jaffe DA (2013) *Effects of short-term macronutrient redistribution on endocrine and performance parameters in resistance trained males*. Master's Thesis, Springfield College, USA.

Jaffe DA, Hewit JK, Sawyer JC, et al. (2018) Effects of short-term macronutrient redistribution on performance parameters in resistance trained males. *Biomedical Journal of Scientific and Techincal Research* 9(2).

Joy J (2017) Determining the ergogenic effects of Carb10™ supplementation on carbohydrate-rich and carbohydrate-restricted diets. Available at: https://clinicaltrials.gov/ct2/show/record/NCT03231514 (accessed 2 August 2021).

Kirwan JP, Costill DL, Mitchell JB, et al. (1988) Carbohydrate balance in competitive runners during successive days of intense training. *Journal of Applied Physiology (1985)* 65(6): 2601–2606.

Kirwan JP, Costill DL, Houmard JA, et al. (1990) Changes in selected blood measures during repeated days of intense training and carbohydrate control. *International Journal of Sports Medicine* 11(5): 362–366.

Krings BM (2018) *The importance of carbohydrate timing during high-intensity training while consuming a low carbohydrate diet*. PhD Thesis, Mississippi State University, USA.

Krings BM, Waldman HS, Shepherd BD, et al. (2021) The metabolic and performance effects of carbohydrate timing in resistance trained males undergoing a carbohydrate restricted diet. *Applied Physiology, Nutrition, and Metabolism* 46(6): 626–636.

Lane A (2009) *The effect of carbohydrate consumption during intensive exercise training on the free testosterone to cortisol ratio*. Master's Thesis, University of North Carolina at Chapel Hill, USA.

Lane AR, Duke JW and Hackney AC (2010) Influence of dietary carbohydrate intake on the free testosterone: cortisol ratio responses to short-term intensive exercise training. *European Journal of Applied Physiology* 108(6): 1125–1131.

Langfort J, Pilis W, Zarzeczny R, et al. (1994) Effect of ketogenic diet on metabolic and hormonal responses to graded exercise in men. *Clinical Science* 87(s1): 35.

Langfort J, Pilis W, Zarzeczny R, et al. (1996) Effect of low-carbohydrate-ketogenic diet on metabolic and hormonal responses to graded exercise in men. *Journal of Physiology and Pharmacology* 47(2): 361–371.

Langfort JL, Zarzeczny R, Nazar K, et al. (2001) The effect of low-carbohydrate diet on the pattern of hormonal changes during incremental, graded exercise in young men. *International Journal of Sport Nutrition and Exercise Metabolism* 11(2): 248–257.

Lima-Silva AE, Pires FO, Bertuzzi RCM, et al. (2011) Low carbohydrate diet affects the oxygen uptake on-kinetics and rating of perceived exertion in high intensity exercise. *Psychophysiology* 48(2): 277–284.

Ludwig D (2014) Framingham state food study (FS 2). Available at: https://clinicaltrials.gov/ct2/show/NCT02068885 (accessed 20 September 2021).

McKay AKA (2020) *The effect of dietary manipulation on iron metabolism and the immune system in elite athletes*. PhD Thesis, The University of Western Australia, Australia.

Mensink M (2019) The effect of a ketogenic diet on the exercise induced immune response. Available at: https://clinicaltrials.gov/ct2/show/study/NCT04019730 (accessed 31 July 2021).

Michalczyk MM, Chycki J, Zajac A, et al. (2019) Anaerobic performance after a low-carbohydrate diet (LCD) followed by 7 days of carbohydrate loading in male basketball players. *Nutrients* 11(4): 778.

Mifflin MD, St Jeor ST, Hill LA, et al. (1990) A new predictive equation for resting energy expenditure in healthy individuals. *The American Journal of Clinical Nutrition* 51(2): 241–247.

Mirtschin JG, Forbes SF, Cato LE, et al. (2018) Organization of dietary control for nutrition-training intervention involving periodized carbohydrate availability and ketogenic low-carbohydrate high-fat diet. *International Journal of Sport Nutrition and Exercise Metabolism* 28(5): 480–489.

Mitchell JB, Pizza FX, Paquet A, et al. (1998) Influence of carbohydrate status on immune responses before and after endurance exercise. *Journal of Applied Physiology (1985)* 84(6): 1917–1925.

Paoli A (2020) Effects of ketogenic diet in body builders. Available at: https://clinicaltrials.gov/ct2/show/NCT04629365 (accessed 31 July 2021).

Paoli A, Cenci L, Pompei P, et al. (2021) Effects of two months of very low carbohydrate ketogenic diet on body composition, muscle strength, muscle area, and blood parameters in competitive natural body builders. *Nutrients* 13(2): 374.

Public Health England (2016) Government dietary recommendations. Report, Public Health England, UK, August.

Rauch JT, Silva JE, Lowery RP, et al. (2014) The effects of ketogenic dieting on skeletal muscle and fat mass. *Journal of the International Society of Sports Nutrition* 11(Suppl 1): P40.

Reed MJ, Cheng RW, Simmonds M, et al. (1987) Dietary lipids: an additional regulator of plasma levels of sex hormone binding globulin. *The Journal of Clinical Endocrinology and Metabolism* 64(5): 1083–1085.

Remer T and Manz F (1994) Estimation of the renal net acid excretion by adults consuming diets containing variable amounts of protein. *The American Journal of Clinical Nutrition* 59(6): 1356–1361.

Remer T, Pietrzik K and Manz F (1998) Short-term impact of a lactovegetarian diet on adrenocortical activity and adrenal androgens. *The Journal of Clinical Endocrinology and Metabolism* 83(6): 2132–2137.

Shaw DM, Merien F, Braakhuis A, et al. (2019) Effect of a ketogenic diet on submaximal exercise capacity and efficiency in runners. *Medicine and Science in Sports and Exercise* 51(10): 2135–2146.

Shaw DM, Merien F, Braakhuis A, et al. (2021) Adaptation to a ketogenic diet modulates adaptive and mucosal immune markers in trained male endurance athletes. *Scandinavian Journal of Medicine and Science in Sports* 31(1): 140–152.

Silva J (2014) The effects of very high fat, very low carbohydrate diets on safety, blood lipid profile, and anabolic hormone status. *Journal of the International Society of Sports Nutrition* 11(1): 1.

Sims EA, Goldman RF, Gluck CM, et al. (1968) Experimental obesity in man. *Transactions of the Association of American Physicians* 81: 153–170.

Sims EA, Bray GA, Danforth EJ, et al. (1974) Experimental obesity in man. VI. the effect of variations in intake of carbohydrate on carbohydrate, lipid, and cortisol metabolism. *Hormone and Metabolic Research = Hormon- und Stoffwechselforschung = Hormones et Metabolisme* Suppl 4: 70–77.

Terink R, Witkamp RF, Hopman MTE, et al. (2021) A 2 week cross-over intervention with a low carbohydrate, high fat diet compared to a high carbohydrate diet attenuates exercise-induced cortisol response, but not the reduction of exercise capacity, in recreational athletes. *Nutrients* 13(1): 157.

Thorp JW, Mittleman KD, Haberman KJ, et al. (1990) Work enhancement and thermal changes during intermittent work in cool water after carbohydrate loading. Report, Naval Medical Research Institute, USA, March.

Toma K (2009) *Effects of high-carbohydrate and low-fat versus high-protein and low-carbohydrate diets on high-intensity aerobic exercise*. PhD Thesis, Ohio University, USA.

Tsai L, Karpakka J, Aginger C, et al. (1993) Basal concentrations of anabolic and catabolic hormones in relation to endurance exercise after short-term changes in diet. *European Journal of Applied Physiology and Occupational Physiology* 66(4): 304–308.

Volek JS, Gómez AL and Kraemer WJ (2000) Fasting lipoprotein and postprandial triacylglycerol responses to a low-carbohydrate diet supplemented with n-3 fatty acids. *Journal of the American College of Nutrition* 19(3): 383–391.

Volek JS, Gómez AL, Love DM, et al. (2001) Effects of a high-fat diet on postabsorptive and postprandial testosterone responses to a fat-rich meal. *Metabolism* 50(11): 1351–1355.

Volek JS, Sharman MJ, Love DM, et al. (2002) Body composition and hormonal responses to a carbohydrate-restricted diet. *Metabolism* 51(7): 864–870.

Waldman HS, Krings BM, Basham SA, et al. (2018) Effects of a 15-day low carbohydrate, high-fat diet in resistance-trained men. *Journal of Strength and Conditioning Research* 32(11): 3103–3111.

Waldman HS, Smith JW, Lamberth J, et al. (2019) A 28-day carbohydrate-restricted diet improves markers of cardiometabolic health and performance in professional firefighters. *Journal of Strength and Conditioning Research* 33(12): 3284–3294.

Waldman HS, Smith JW, Lamberth J, et al. (2020) A 28-day carbohydrate-restricted diet improves markers of cardiovascular disease in professional firefighters. *Journal of Strength and Conditioning Research* 34(10): 2785–2792.

Wang C, Catlin DH, Starcevic B, et al. (2005) Low-fat high-fiber diet decreased serum and urine androgens in men. *The Journal of Clinical Endocrinology and Metabolism* 90(6): 3550–3559.

Werner TJ (2006) *The effect of high-carbohydrate, low-fat & low-carbohydrate, high protein diets on physiologic and performance variables on row ergometry training*. Master's Thesis, Ohio University, USA.

Whittaker J and Harris M (2021) Low carbohydrate diets and men’s testosterone and cortisol: systematic review and meta-analysis. Available at: https://www.crd.york.ac.uk/prospero/display_record.php?RecordID=255957 (accessed 18 August 2021).

Wilson JM, Lowery RP, Roberts MD, et al. (2020) Effects of ketogenic dieting on body composition, strength, power, and hormonal profiles in resistance training men. *Journal of Strength and Conditioning Research* 34(12): 3463–3474.

Witt KA, Snook JT, O’Dorisio TM, et al. (1993) Exercise training and dietary carbohydrate: effects on selected hormones and the thermic effect of feeding. *International Journal of Sport Nutrition* 3(3): 272–289.

Zajac A, Poprzecki S, Maszczyk A, et al. (2014) The effects of a ketogenic diet on exercise metabolism and physical performance in off-road cyclists. *Nutrients* 6(7): 2493–2508.
